# The Protective Effect of Pilose Antler Peptide on CUMS-Induced Depression Through AMPK/Sirt1/NF-κB/NLRP3-Mediated Pyroptosis

**DOI:** 10.3389/fphar.2022.815413

**Published:** 2022-03-23

**Authors:** Yue Hu, Min Zhao, Tong Zhao, Mingming Qi, Guangda Yao, Yu Dong

**Affiliations:** ^1^ School of Chinese Medicine, School of Integrated Chinese and Western Medicine, Nanjing University of Chinese Medicine, Nanjing, China; ^2^ School of Pharmacy, Nanjing University of Chinese Medicine, Nanjing, China

**Keywords:** pilose antler peptide, CUMS, AMPK, SIRT1, NF-κB, NLRP3, pyroptosis

## Abstract

**Background:** Pilose antler peptide (PAP), prepared from the pilose antler of *Cervus nippon* Temminck, is widely used in traditional Chinese medicine (TCM) against various inflammatory disorders. TCM prescriptions containing pilose antler are often prescribed clinically to treat depression. However, the pharmacological mechanisms of how PAP, against inflammation, prevents and treats depression remain poorly understood.

**Methods:** PAP was identified by *de novo* sequencing and database searching. Then, behavioral tests were conducted to investigate the effect of PAP on CUMS-exposed mice. In parallel, Nissl staining and Golgi-Cox staining were used for exploring the effect of PAP on neural cells and dendritic spine density. Additionally, the expression of key proteins of the AMPK/Sirt1/NF-κB/NLRP3 pathway was analyzed by Western blot. Finally, the CUMS procedure was conducted for 6 weeks. At the 5th week, PAP and fluoxetine (Flu) were intragastrically treated for 2 weeks. The silencing information regulator-related enzyme 1 (Sirt1) inhibitor EX-527 and the AMP-activated protein kinase (AMPK) inhibitor dorsomorphin were employed to investigate the effects of Sirt1 and AMPK on PAP-mediated depression.

**Results:** PAP attenuated the behavior alteration caused by CUMS stimulation, decreased the number of neurons, and restored the dendritic spine density. PAP treatment effectively upregulated the expressions of p-AMPK and Sirt1 and suppressed the expressions of Ac-NF-κB, NLRP3, Ac-Caspase-1, GSDMD-N, Cleaved-IL-1β, and Cleaved-IL-18. Moreover, selectively inhibited Sirt1 and AMPK were able to compromise the therapeutic effect of PAP on depression.

**Conclusion:** The present work indicated that PAP has a protective effect on CUMS-induced depression. In addition, AMPK and Sirt1 played critical roles in the PAP-relieved depression. PAP might be a potential therapeutic option for treating depression.

## Introduction

Depression, as a common emotional disorder, affects more than 300 million people worldwide, causing nearly 800,000 suicides every year (Malhi and Mann, 2018), thus bringing a heavy burden to individuals and the society (GBD 2016 Disease and Injury Incidence and Prevalence Collaborators, 2018). A few drugs have been explored for the intervention of depression. Unfortunately, they were not satisfied in the clinic due to their side effects including gastrointestinal symptoms or sexual disorders (Schramm et al., 2020). For instance, fluoxetine hydrochloride (Flu), known as selective serotonin reuptake inhibitors (SSRIs), has been approved for the treatment of depression by the United States FDA for over 30 years (Wong et al., 2005). Although a proper dose of Flu usually has no serious toxic effects, several side effects can be seen in patients commonly, e.g., nausea, anorexia, insomnia, and nervousness (Gram, 1994; Jakubovski et al., 2016). Therefore, there is an urgent need for discovering the potential candidate compounds.

Researchers around the world are trying to study the underlying mechanisms of depression. It is speculated that imbalanced neurotransmitters, excessive oxidative stress, and maladjusted apoptosis aggravate the development of depression (Otte et al., 2016). Currently, pyroptosis has attracted more and more attention to the development of neuro-dysfunction (Van Opdenbosch and Lamkanfi, 2019). Pyroptosis is a kind of pro-inflammatory programmed cell death featured by the assembly and activation of the NLRP3 inflammasome (Schroder and Tschopp, 2010). The activation of the NLRP3 inflammasome requires the recruitment of ASC and the auto-activation of Caspase-1 into cleaved Caspase-1, which promote the activation of GSDMD and its cleavage to form GSDMD-N (Liu et al., 2020). It is generally acknowledged that the transcriptional factor NF-κB drives the activation of the NLRP3 inflammasome in the pyroptosis procession (Lei et al., 2018). Silencing information regulator-related enzyme 1 (Sirt1), an NAD^+^-dependent deacetylase, possesses multiple functions in DNA repair, transcription recombination, stress resistance, oxidative stress, inflammation, and apoptosis (Rada et al., 2018; Feng et al., 2019; Meng et al., 2020). The acetylation of NF-κB was governed by Sirt1 in an inflammatory reaction (Peng et al., 2019). Moreover, AMP-activated protein kinase (AMPK) is a key modulator of cellular energy metabolism. It was demonstrated that AMPK regulated the phosphorylation of NLRP3-mediated pyroptosis *via* kinases or phosphatases in macrophages (Deng et al., 2020). However, the role of AMPK/Sirt1 as a regulator of NLRP3-mediated pyroptosis has not been explained.

Pilose antler, namely “lu rong”, comes from the soft growing tissues of *Cervus nippon* Temminck and is a common aphrodisiac in traditional Chinese medicine. Pilose antler peptide (PAP) is extracted and purified from pilose antler. It has been recently reported that PAP exerts various properties including renal-protective, neuroprotective, anti-aging, anti-oxidative, and anti-inflammatory activities (Chunhui et al., 2017; Dong et al., 2018; Wu et al., 2018; Zhao et al., 2019; Yun et al., 2020). It was proposed that PAP exhibited a beneficial effect on cerebral ischemia/reperfusion (I/R) injury (Bai et al., 2017). PAP also showed anti-apoptotic properties against sevoflurane-mediated neurocyte injury (Li and He, 2018). Nevertheless, whether PAP can treat the depressive disorder is unclear. The present work was conducted to evaluate the pharmacological effect of PAP on CUMS-induced depression and explore its potential mechanism.

## Materials and Methods

### Reagents

Fluoxetine hydrochloride (Flu) produced by Tokyo Chemical Industry Co., Ltd. (Tokyo, Japan) was dissolved in a saline solution. The Sirt1 inhibitor EX-527 (#HY-15452) and AMPK inhibitor dorsomorphin (#HY-13418A) were purchased from MedChemExpress (Shanghai, China). All the antibodies were purchased from Cell Signaling Technology (Danvers, Massachusetts, United States) or Abcam (Cambridge, United Kingdom). Trypsin solution for nano LC-LTQ Orbitrap analysis was purchased from Promega Co., Ltd. (Fitchburg, United States). Ethanol, acetonitrile, formic acid, and glacial acetic acid of HPLC grade were obtained from Merck Co., Ltd. (Darmstadt, Germany). The slices of pilose antler were purchased from Linguanlu company (Jilin, China).

### The Extraction and Preparation of Pilose Antler Peptide

The slices of pilose antler were meticulously grinded into pilose antler powder. Then, a weight of 0.2 g pilose antler powder was dissolved into pure water (40 times amount of the powder). After centrifugation for 10 min at 12,000× G at 4°C, the power solution was lyophilized and stored at 4°C for use. Every time before experiments, the pilose antler lyophilized powder was dissolved into 200 μl of 0.2% formic acid solution. After centrifugation for 10 min at 21,000× G at 4°C, the supernatant was collected and removed in a 3-KD ultrafiltration centrifuge tube and then centrifuged for 30 min at 14,000× G. Afterward, the lower liquid was collected and lyophilized. Most of the lower solution was composed of peptides. The final PAP was dissolved in DMSO. In all experiments, the final concentration of DMSO was less than 0.1% (v/v).

### Nano LC-LTQ Orbitrap Analysis

For an accurate mass measurement, an LTQ Orbitrap Velos mass spectrometer (Thermo Fisher, United States) equipped with the UltiMate 3000 RSLCnano system (Dionex, California, United States) was used. First, samples of proteins were digested with trypsin (Fitchburg, United States). The filtered pilose antler polypeptide sample was analyzed by using a nano-trap column (Acclaim PepMap^®^ RSLC C_18_, 75 μm × 150 mm, 5 μm) under the gradient elution from 5 to 40% (v/v) acetonitrile containing 0.1% formic acid over 150 min at a flow rate of 300 nl/min. The mass range was from m/z 300 to 1800. Data file analysis was performed by PEAKS Studio 8.5 software (Bioinformatics Solutions Inc., Canada).

### Animals

All procedures on animals were approved by the Committee on the Ethics of Animal Experiments of the Nanjing University of Traditional Chinese Medicine. Male and female Balb/c mice (10 weeks old, weighted 18–22 g) were obtained from Kawensi Co., Ltd (Changzhou, China). The animals were maintained under standard conditions for 1 week of adaptation. The mice were housed at 23 ± 2°C under a 12 h light/12 h dark cycle, with free access to food and water. All procedures were carried out in accordance with the principles for laboratory animals (NIH publication #85-23, revised in 1985).

### Randomization and Blinding

For each series of experiments, mice were assigned randomly to each group by drawing lots. The data analysis was performed by a researcher blinded with respect to the treatment.

### CUMS Procedure and Experimental Design

To evaluate the effect of PAP on CUMS-induced mice and further investigate its mechanism, three series of experiments with twelve mice in each group were conducted. For experiment 1, the mice were randomly assigned to the Control group, CUMS group, CUMS + PAP (50 mg/kg) group, CUMS + PAP (100 mg/kg) group, and CUMS + Flu group. For experiment 2, the mice were randomly divided into the CUMS + Vehicle + normal saline group, CUMS + EX-527 + normal saline group, CUMS + Vehicle + PAP group, and CUMS + EX-527 + PAP group. For experiment 3, the mice were randomly divided into the CUMS + Vehicle + normal saline group, CUMS + Dorsomorphin + normal saline group, CUMS + Vehicle + PAP group, and CUMS + Dorsomorphin + PAP group.

After the adaptation, all mice were subjected to the CUMS procedure except for the control group. The 6-week CUMS-induced treatment was performed according to the previously described method with minor modifications (Xue et al., 2015). The mice were subjected to discontinuously random mild stress including food and water deprivation, overnight illumination, moist bedding, 45°C cage tilting, light/dark succession, and rat-hole scabbard constriction. The overnight illumination was performed twice or three times per week. From the 28th day, PAP (50 mg/kg), PAP (100 mg/kg), and Flu (20 mg/kg) were treated intragastrically once a day for 2 weeks. The mice in the control group and CUMS group were treated with normal saline simultaneously. Afterward, the depressive symptoms were evaluated by the behavior test, and then, the mice were sacrificed. In experiments 2 and 3, from the 28th day, the mice were intravenously injected at a dose of 10 mg/kg EX-527 or 10 mg/kg dorsomorphin for 2 weeks.

### Sucrose Preference Test

The sucrose preference test is a commonly used method to assess anhedonia that served as the core symptom of depression. After adaption to 1% sucrose solution, each mouse was housed separately in this test. After 24 h deprivation of food and water, each mouse was given two bottles containing tap water or 1% sucrose solution. 1 h later, the bottle was weighed, and the consumption was recorded. The sucrose preference ratio was calculated according to the following formula: sucrose solution consumption/(tap water consumption + sucrose solution consumption).

### Open Field Test

In order to assess the locomotor activity, the OFT was performed using the opaque plexiglass-made (100 × 100 × 40 cm) box in a quiet environment. The apparatus was virtually divided into 25 equal sectors. During the test, every mouse was gently placed into the center of the apparatus and permitted to explore freely for 5 min. Thereafter, the number of distances traveled (with all four paws placed into a new square) and time in the center were monitored by ANY-maze software. The apparatus was cleaned with 75% ethanol after each mouse was tested.

### Forced Swimming Test

The mice were placed in an open cylindrical plexiglass container (20 cm in height and 14 cm in diameter) complemented with 10 cm-deep water (25 ± 1°C). The mice were forced to swim for 6 min, in which the immobile time during the final 4 min was recorded by two observers who were blinded to the group assignment. A mouse that floats in the water without struggling was defined as stationary. After the test, the mice were immediately dried with air conditioner and returned to cages.

### Tail Suspension Test

The TST was conducted by using a computerized system to evaluate the antidepressant activity of PAP. The mice were taped about 1 cm from the tip of their tails and hung about 20 cm from the floor. The experiment was performed for 6 min, in which the total immobility duration was recorded within the last 4 min.

### Nissl Staining

After the behavioral tests, three mice from each group were anesthetized and perfused with 4% paraformaldehyde through the left heart ventricle. The brain tissues were collected and fixed immediately in 4% paraformaldehyde for 48 h. The paraffin-embedded hippocampi were cut into 4 mm slides. The slides were dehydrated in gradient ethanol, immersed in xylene, and rehydrated in descending grades of ethanol. The slides were hydrated in 1% toluidine blue at 60°C for 30 min, dehydrated in ethanol, removed in xylene, and sealed with neutral gum. The hippocampal morphology was observed under a light microscope (DM1000, Leica, Germany).

### Golgi-Cox Staining of Dendritic Spines

The Golgi-Cox staining was carried out using the super Golgi Kit (Bioenno Tech, Irvine, CA, United States). The mice were anesthetized and perfused with 4% paraformaldehyde. The brains were harvested and maintained in the Golgi solution in dark for 3 days at 37°C. After dehydration in 30% sucrose solution, the brain tissues were embedded in 4% agar. The sample was cut into slices. After 24 h, the sections were dehydrated with 50% ethanol, ammonia, and sodium thiosulfate. The sections were treated with gradient ethanol and xylene and then sealed with neutral balsam. Images were analyzed under an Olympus focal fluorescent microscope (IX73, Olympus, Japan).

### Western Blot

The proteins of hippocampus samples were extracted with a protein extraction solution containing 1% protease inhibitors and 1% phosphatase inhibitors and centrifuged at 12,000 g for 20 min. The protein concentration in the supernatant was quantified by BCA protein assay (Beyotime, Shanghai, China). For the test, 8–12% SDS-PAGE gel was applied to separate proteins. The sample was electrotransferred onto the PVDF membrane and blocked in a 5% milk solution. Then, the membrane was incubated with primary antibodies at 4°C overnight as following: anti-p-AMPK (#2537S, 1:1000), anti-Sirt1 (#8469S, 1:1000), anti-Ac-NF-κB (#3033S, 1:1000), anti-NF-κB (#8242S, 1:1000), anti-NLRP3 (#15101S, 1:1000), anti-ASC (#67824S, 1:1000), anti-Ac-Caspase-1 (#89332S, 1:1000), anti-Caspase 1 (#24232S, 1:1000), anti-GSDMD-N (#93709S, 1:1000), anti-Cleaved IL-Iβ (#52718S, 1:1000), and anti- Cleaved IL-18 (#PB0058, 1:500). After rinsing three times, the membrane was incubated with an appropriate horseradish peroxidase-conjugated secondary antibody at room temperature for 1.5 h. Consequently, the protein bands were detected by using an enhanced chemiluminescence system and analyzed by ImageJ software.

### Immunofluorescence Analysis

The immunofluorescence analysis was performed when mice were sacrificed after the behavioral tests ended. Brains were perfused with 4% paraformaldehyde. Afterward, samples were sliced through the whole hippocampus by cryostat (Leica CM 1950) and then blocked with 3% bovine serum albumin (BSA) at room temperature. Samples were incubated with primary antibodies as follows: Phospho-AMPKα (Thr172) rabbit mAb (CST, #2535, 1:1000) and NLRP3 monoclonal antibody (Invitrogen, #768319, 1:1000). Afterward, the sections were exposed to Alexa Fluor 594 or Alexa Fluor 488-conjugated secondary antibodies and further counterstained for approximately 15 min with 4′-6-Diamidino-2-phenylindole (DAPI) solution. Immunofluorescent images were analyzed with a confocal laser scanning microscope (Olympus).

### Statistical Analysis

All data are presented as mean ± SD. The results were statistically analyzed by one-way ANOVA with Tukey’s multiple comparison tests using GraphPad 5.0 software. A value of *p* < 0.05 was identified as statistically significant, and a value of *p* < 0.01 was identified extremely significant.

## Results

### Identification of Pilose Antler Peptide

The total ion flow chromatogram (TIC) consists of two parts: the peptide part with a molecular weight less than 3 KD (Supplementary Figure S1) and the protein with a molecular weight greater than 3 KD. The mall peptides with a molecular weight of less than 3 KD were sequenced by mass spectrometry. The *de novo* sequencing and database searching were used to identify the peptides in pilose antler. The peptides with an ALC (average local confidence) score greater than 80 were selected as sequencing trusted peptides. A total number of 12 peptides were identified in pilose antler. The information of each peptide is shown in Table 1.

**TABLE 1:** Identified peptides in pilose antler.

No.	RT (min)	m/z	Molecular weight	Error (ppm)	Length	ALC (%)	Polypeptide sequence
1	34.48	555.782 6	1109.546 6	3.6	11	89	LPNSSATLGNH
2	40.78	548.278 7	1094.564 7	−19.9	10	80	LSALEGVFYP
3	42.01	551.785 2	1101.548 8	6.4	10	82	MEKLNGGKEP
4	42.54	408.707 3	815.396 6	4.2	6	85	DHLVWF
5	42.92	585.840 4	1169.665 5	0.6	10	85	LVLVEAELRE
6	43.73	406.192 3	810.358 8	13.8	6	93	TYFYAF
7	48.29	562.244 4	1122.462 6	10.3	9	81	FGFDLCCYR
8	48.41	529.769 9	1057.519 3	5.6	9	81	DHNAKEVVF
9	50.08	388.192 2	774.373 4	−4.7	6	97	FVEHML
10	52.11	584.804 3	1167.592 3	1.5	10	82	LDLSFFNQGK
11	52.31	638.849 4	1275.693 6	−7.3	13	84	APLLDGGNAALKH
12	55.48	813.377 0	1624.752 2	−7.9	14	82	AFQGYNAVDDLLHY

### The Effect of Pilose Antler Peptide on Depression-Like Behaviors

**FIGURE 1:**
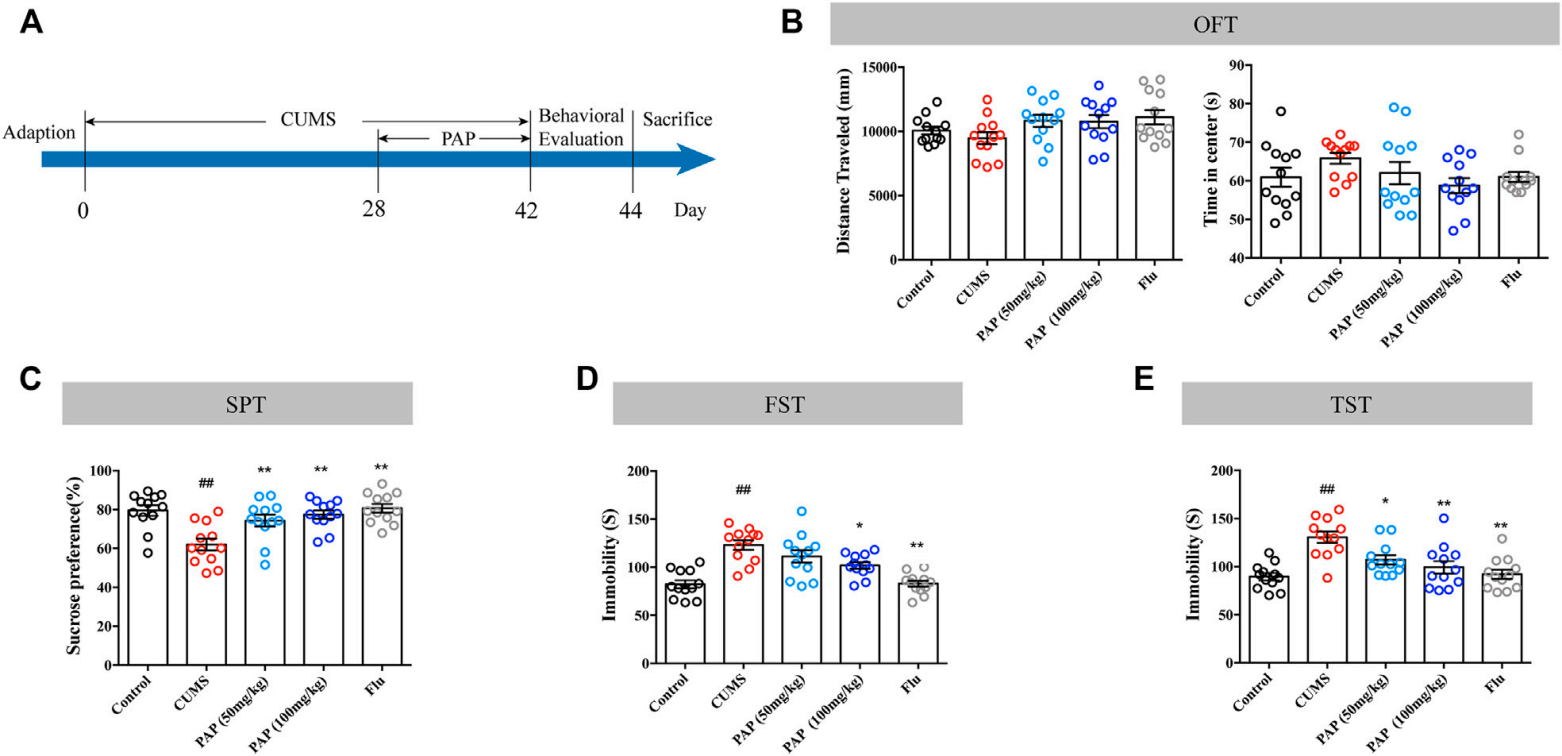
Effect of PAP on depression-like behaviors. The schedule of CUMS induction and drug treatment in experiment 1 **(A)**. The distance traveled and time in the center of the OFT **(B)**. The sucrose preference in the SPT **(C)**. The immobility in the FST **(D)** and TST **(E)**. ##*p* < 0.01 vs. control group, *p < 0.05, and **p < 0.01 vs. CUMS group.

The timeline of the first experimental series investigating the effect of PAP is shown in Figure 1A. The OFT, SPT, FST, and TST were performed to evaluate the effect of PAP on depression-like behaviors. As illustrated in Figure 1B, CUMS stimulation did not affect the distance traveled and time in the center compared with those in the Control group. The treatment with PAP (50 mg/kg), PAP (100 mg/kg), and Flu also did not cause significant differences compared with those in the CUMS group. The data of the OFT indicated that PAP did not influence the locomotor activity of mice.

Compared with the Control group, exposure to CUMS contributed to the decreased sucrose preference (Figure 1C, *p* < 0.01). The administration of PAP (50 mg/kg), PAP (100 mg/kg), and Flu showed an increase in sucrose consumption compared with those in the CUMS group (Figure 1C, *p* < 0.01).

Additionally, the CUMS challenge notably increased the immobile duration in the FST and TST (Figures 1D,E, *p* < 0.01). By contrast, the treatment of PAP (100 mg/kg) (*p* < 0.05) and Flu (*p* < 0.01) evidently decreased the immobility than that in the CUMS group in the FST. During the TST experiment, the administrations of PAP (100 mg/kg) (*p* < 0.01), PAP (50 mg/kg) (*p* < 0.05), and Flu (*p* < 0.01) notably reduced the immobile duration compared with that in the CUMS group.

### The Effect of Pilose Antler Peptide on the Nissl Body and Dendritic Spine Density

**FIGURE 2:**
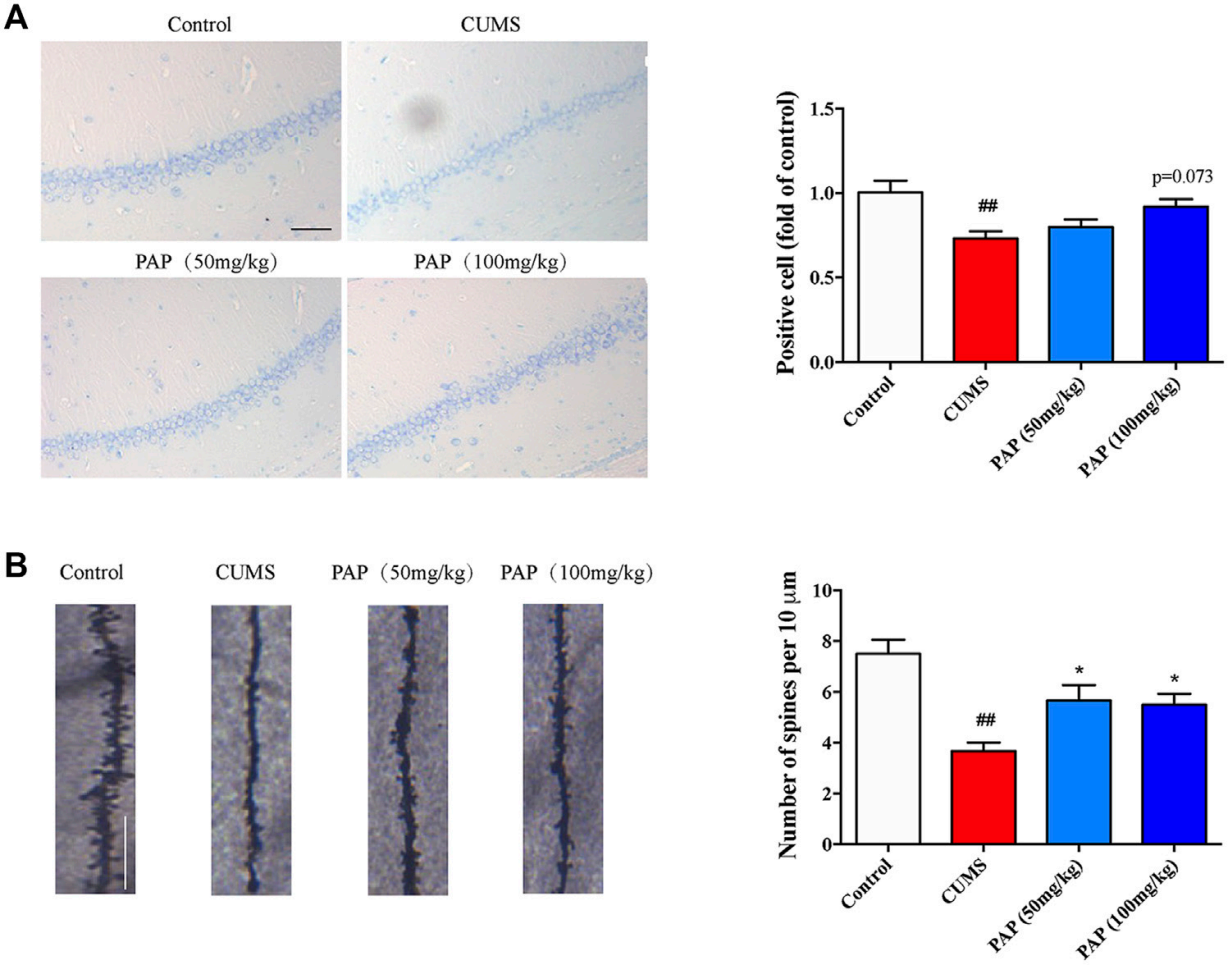
Effect of PAP on the Nissl body and dendritic spine density. The positive cell in Nissl staining **(A)** and the number of spines per 10 μm^2^ **(B)** of Golgi-Cox staining. ##*p* < 0.01 vs. control group and *p < 0.05 vs. CUMS group. Scale bar: 100 μm **(A)**, 10 μm **(B)**.

Nissl staining was used to observe the histological changes of the CA1 region in CUMS-exposed mice. The Nissl body is the major component responsible for neuron protein synthesis, which governs the excitation and conduction of neurons. In Nissl staining, the CUMS challenge conduced to the dark staining of positive cells, while in the PAP treatment group, nuclei and abundant Nissl bodies were seen (Mohammadi et al., 2020). In addition, the integrity of the structure and function of dendritic spines are involved in the information transmission of neurons. As shown in Figure 2A, CUMS stimulation remarkably decreased the number of positive cells (*p* < 0.01), whereas the number of positive cells was slightly increased after PAP treatment compared with that in the CUMS group.

Next, we detected the dendritic spine density to evaluate the synaptic transmission and synaptic plasticity (Figure 2B). Exposure to CUMS contributed to the reduction of the dendritic spine density (*p* < 0.01), whereas PAP administration significantly increased the dendritic spine density compared with that in the CUMS group (*p* < 0.05). Dendritic spines are widely damaged in hippocampal neurons of depression patients (Luo et al., 2020). Our observation showed that PAP restored the dendritic spine density in CUMS-induced mice, which indicated the protective effects of PAP on the cortex neurons from the damage induced by CUMS.

### The Effect of Pilose Antler Peptide on the AMPK/Sirt1/NF-κB/NLRP3 Pathway

**FIGURE 3:**
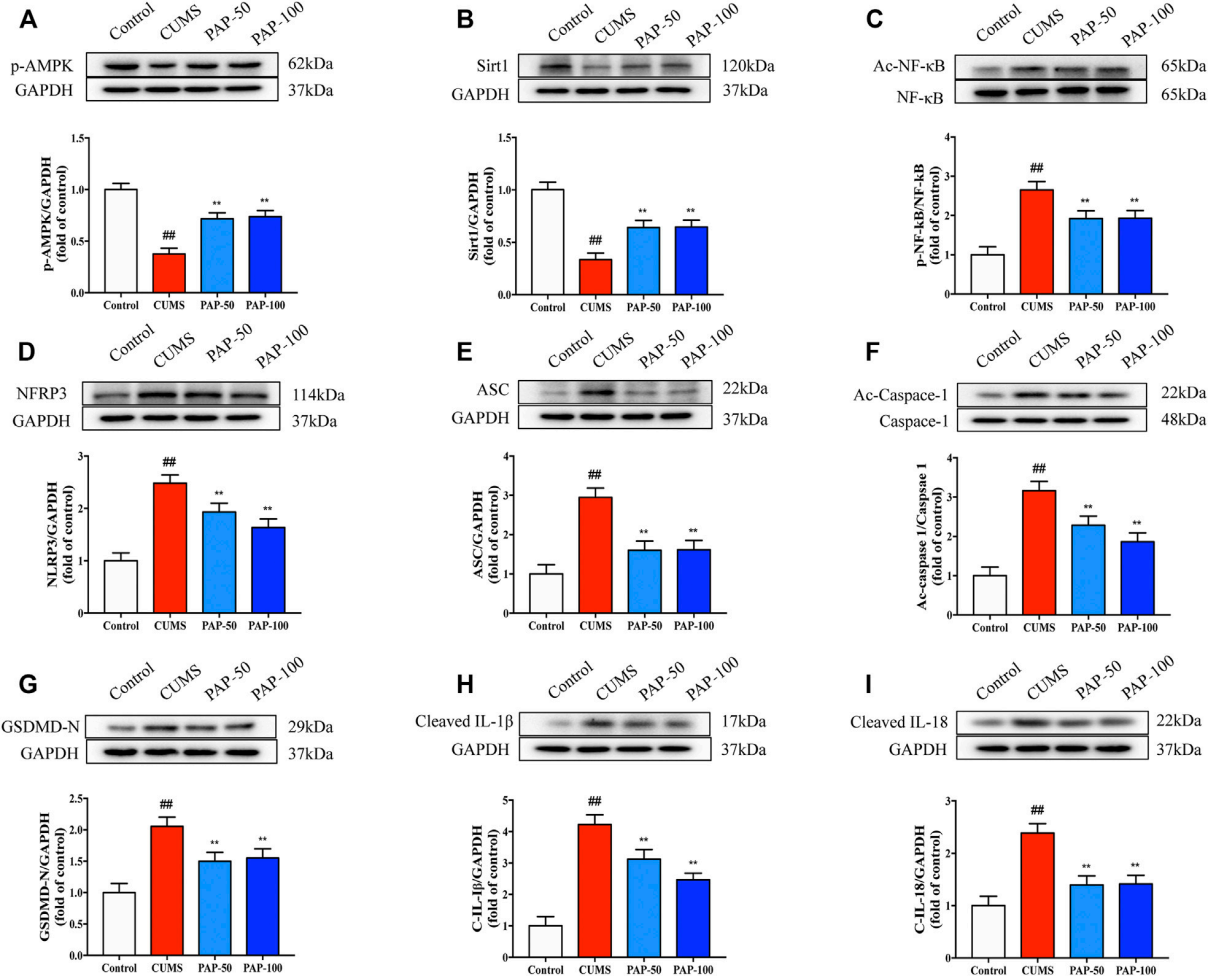
Effect of PAP on the AMPK/Sirt1/NF-κB/NLRP3 pathway. The protein expressions of p-AMPK **(A)**, Sirt1 **(B)**, Ac-NF-κB **(C)**, NLRP3 **(D)**, ASC **(E)**, Ac-Caspase-1 **(F)**, GSDMD-N **(G)**, Cleaved-IL-1β **(H)**, and Cleaved-IL-18 **(I)** in hippocampal tissues. ##*p* < 0.01 vs. control group, *p < 0.05, and **p < 0.01 vs. CUMS group.

To investigate the mechanism involved in the treatment of PAP on CUMS-induced depression, the protein expression levels of p-AMPK, Sirt1, Ac-NF-κB, NF-κB, NLRP3, ASC, Ac-Caspase-1, GSDMD-N, Cleaved-IL-1β, and Cleaved-IL-18 were analyzed by Western blot (Figure 3). CUMS treatment led to a significant downregulation of p-AMPK and Sirt1 and a significant upregulation of Ac-NF-κB, NLRP3, ASC, Ac-Caspase-1, GSDMD-N, Cleaved-IL-1β, and Cleaved-IL-18. PAP treatment effectively restored the expression of p-AMPK and Sirt1 and suppressed that of Ac-NF-κB, NLRP3, Ac-Caspase-1, GSDMD-N, Cleaved-IL-1β, and Cleaved-IL-18 compared with that of the CUMS group. In addition, the result of immunofluorescence showed that the expression of p-AMPK was downregulated in the CUMS model, while NLRP3 was upregulated. However, PAP treatment could increase the expression of p-AMPK and inhibit the expression of NLRP3. Moreover, p-AMPK and NLRP3 were co-localized in cells of the hippocampus (Supplementary Figure S2). The analytical results suggested that PAP exhibited protective effects on CUMS-induced depression possibly through the AMPK/Sirt/NF-κB/NLRP3 signaling pathway.

### Pilose Antler Peptide Attenuated Sirt1 in CUMS-Induced Depression-Like Behaviors

**FIGURE 4:**
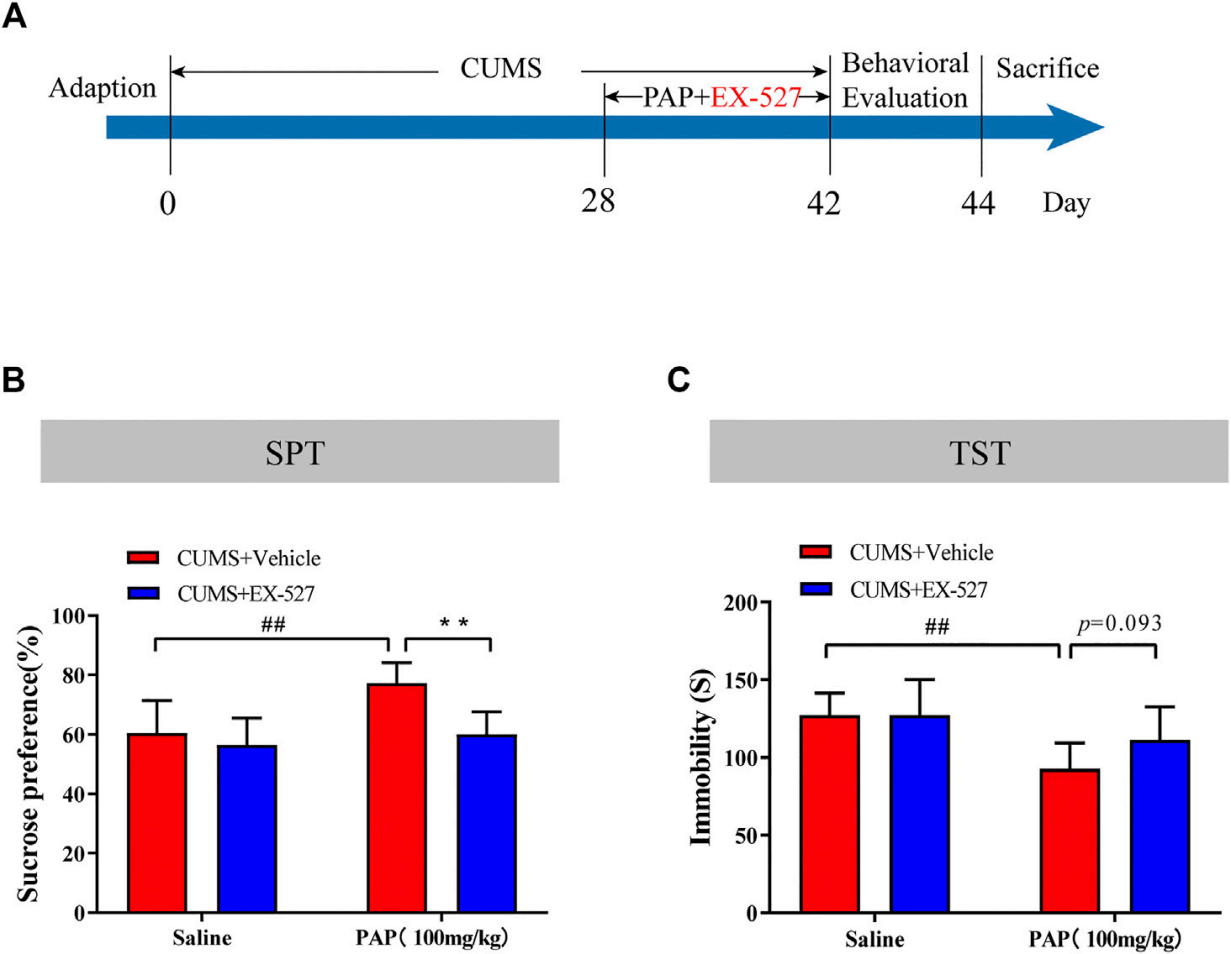
Role of Sirt1 in the attenuation of PAP on CUMS-induced depressive behavior. The schedule of CUMS induction and drug treatment in experiment 2 **(A)**. The sucrose preference in the SPT **(B)**. The immobility in the TST **(C)**. ##*p* < 0.01 vs. CUMS + Vehicle, **p < 0.01 vs. CUMS + Vehicle + PAP group.

The Sirt1 selective inhibitor EX-527 was employed to further study the mechanism of PAP-mediated depression. As a selective Sirt1 inhibitor, EX-527 occupies the nicotinamide site and contacts the ribose of NAD^+^ to exploit Sirtuin/ligand crystal structures (Gertz et al., 2013). The timeline of this series of experiments is shown in Figure 4A. Then, the SPT and TST were performed for detecting the depression-like behaviors in each group. As shown in Figures 4B,C, the sucrose preference in the CUMS + PAP (100 mg/kg) group was significantly increased compared with that in the CUMS + Vehicle group (*p* < 0.01), while the sucrose preference in the CUMS + EX-527 + PAP (100 mg/kg) group was markedly inhibited compared with that in the CUMS + PAP (100 mg/kg) group. Besides, the immobile duration in the CUMS + PAP (100 mg/kg) group was significantly decreased compared with that of the CUMS + Vehicle group (*p* < 0.01). However, the immobile time in the CUMS + EX-527 + PAP (100 mg/kg) group had no significant difference compared with that of the CUMS + EX-527 group. The FST showed the same tendency as the TST (Supplementary Figure S3A). The data indicated that Sirt1 played a critical role in the regulation of PAP-treated depression.

### The Role of Sirt1 in the Attenuation of Pilose Antler Peptide Against CUMS-Induced Depression

**FIGURE 5:**
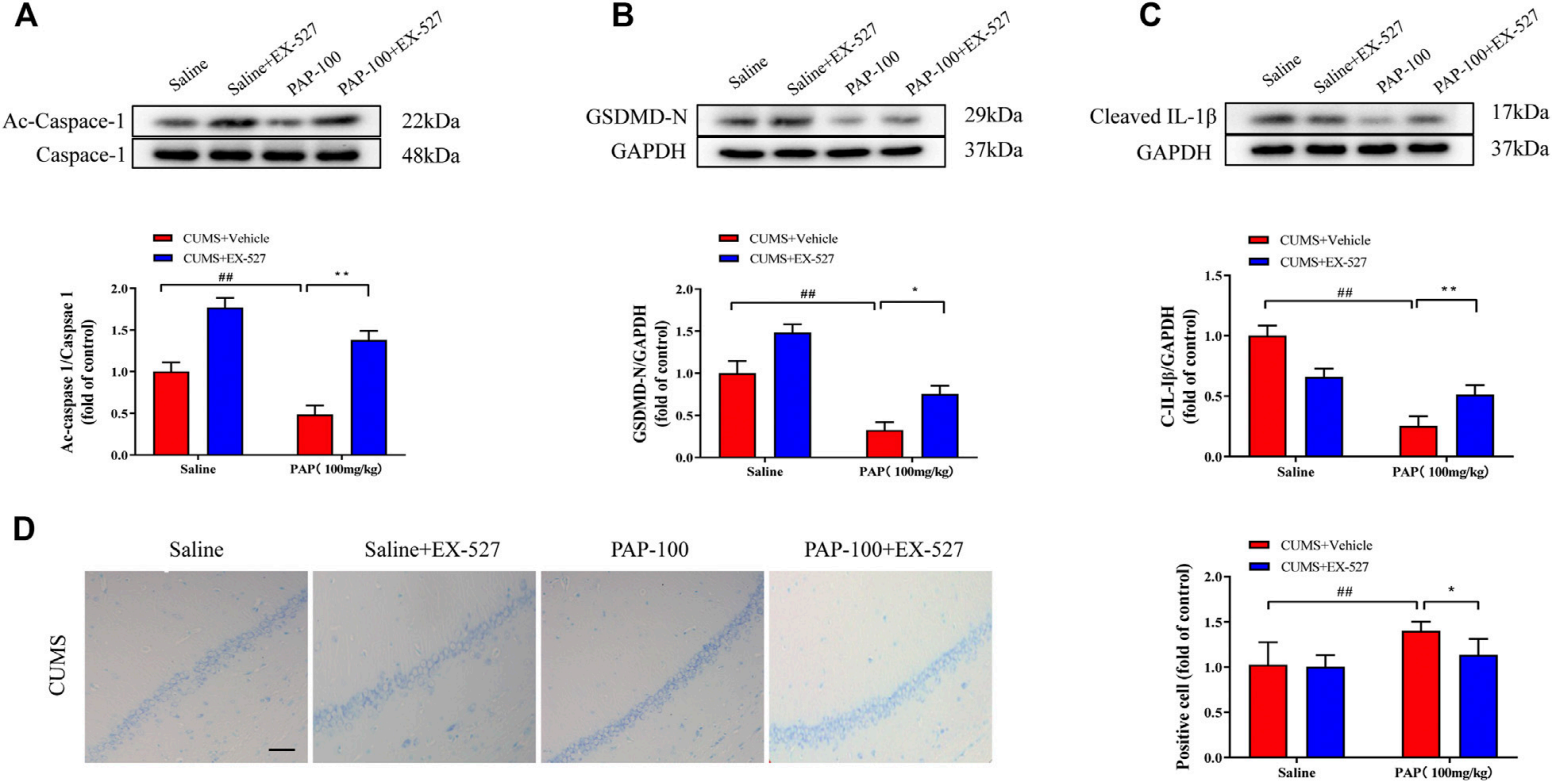
Role of Sirt1 in the attenuation of PAP on CUMS-induced depression. The expressions of Ac-Caspase-1 **(A)**, GSDMD-N **(B)**, and Cleaved-IL-1β **(C)** in hippocampal tissues. The positive cell in Nissl staining **(D)**. ##*p* < 0.01 vs. CUMS + Vehicle, *p < 0.05, and **p < 0.01 vs. CUMS + Vehicle + PAP group. Scale bar: 100 μm.

Next, the expressions of Ac-Caspase-1, GSDMD-N, and Cleaved-IL-1β were detected. As revealed in Figures 5A–C, the protein levels of Ac-Caspase-1, GSDMD-N, and Cleaved-IL-1β were all obviously reduced in the CUMS + PAP (100 mg/kg) group compared with those in the CUMS + Vehicle group (*p* < 0.01). The expression levels of Ac-Caspase-1 (*p* < 0.01) and GSDMD-N (*p* < 0.05) in the CUMS + EX-527 + PAP (100 mg/kg) group were pronouncedly higher than those in the CUMS + PAP (100 mg/kg) group.

Moreover, in Nissl staining, the positive cells in the CUMS + PAP (100 mg/kg) group were dramatically increased compared with those in the CUMS + Vehicle group (*p* < 0.01) (Figure 5D). Nonetheless, the number of Nissl bodies in the CUMS + EX-527 + PAP (100 mg/kg) group notably downregulated compared with that of the CUMS + PAP (100 mg/kg) group (*p* < 0.05). Our results displayed that Sirt1 was involved in the alterations of pyroptosis and the Nissl body in PAP-regulated CUMS-exposed depression.

### The Role of AMPK in the Attenuation of Pilose Antler Peptide Against CUMS-Induced Depression

**FIGURE 6:**
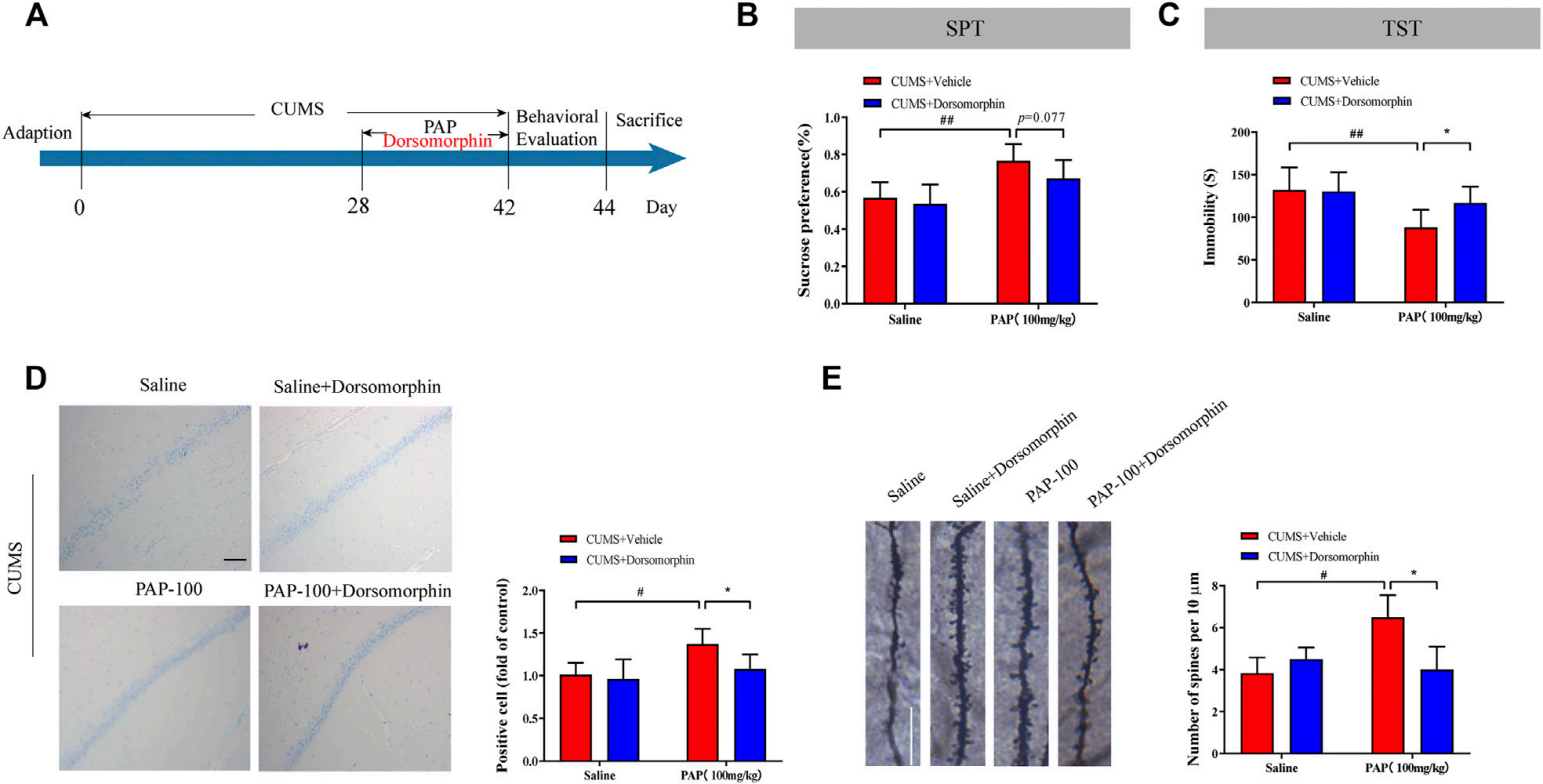
Role of AMPK in the attenuation of PAP on CUMS-induced depression. The schedule of CUMS induction and drug treatment in experiment 3 **(A)**. The sucrose preference in the SPT **(B)**. The immobility in TST **(C)**. The positive cell in Nissl staining **(D)** and the number of spines per 10 μm^2^ **(E)** of Golgi-Cox staining. *p < 0.05, #*p* < 0.05, ##*p* < 0.01 vs. CUMS + Vehicle, and *p < 0.05 vs. CUMS + Vehicle + PAP group. Scale bar: 100 μm **(D)**, 10 μm **(E)**.

Furthermore, the AMPK inhibitor dorsomorphin was employed to investigate the effect of AMPK on PAP-modulated depression (Figure 6A). Dorsomorphin, also known as compound C, is a pyrazolopyrimidine that is wildly used as a cell-permeable AMPK inhibitor (Rembacz et al., 2019). As shown in Figures 6B,C, the enhanced sucrose preference in the SPT and reduced immobile time of the TST in the CUMS + PAP (100 mg/kg) group were observed compared with those in the CUMS + Vehicle (*p* < 0.01) group. In contrast, the immobility in the CUMS + Dorsomorphin + PAP (100 mg/kg) group was inhibited compared with that of the CUMS + PAP (100 mg/kg) group. In addition, reduced immobile time of the FST in the CUMS + PAP (100 mg/kg) group can be reversed by dorsomorphin (*p* < 0.05). All these data indicated that AMPK service is an important regulator in PAP-treated depression (Supplementary Figure S3B).

The Nissl body and dendritic spine density were calculated in different groups. Compared with the CUMS + Vehicle group, the number of positive cells and spines was increased in the CUMS + PAP (100 mg/kg) group (*p* < 0.05) and those were reduced in the CUMS + Dorsomorphin + PAP (100 mg/kg) group (*p* < 0.05).

**FIGURE 7:**
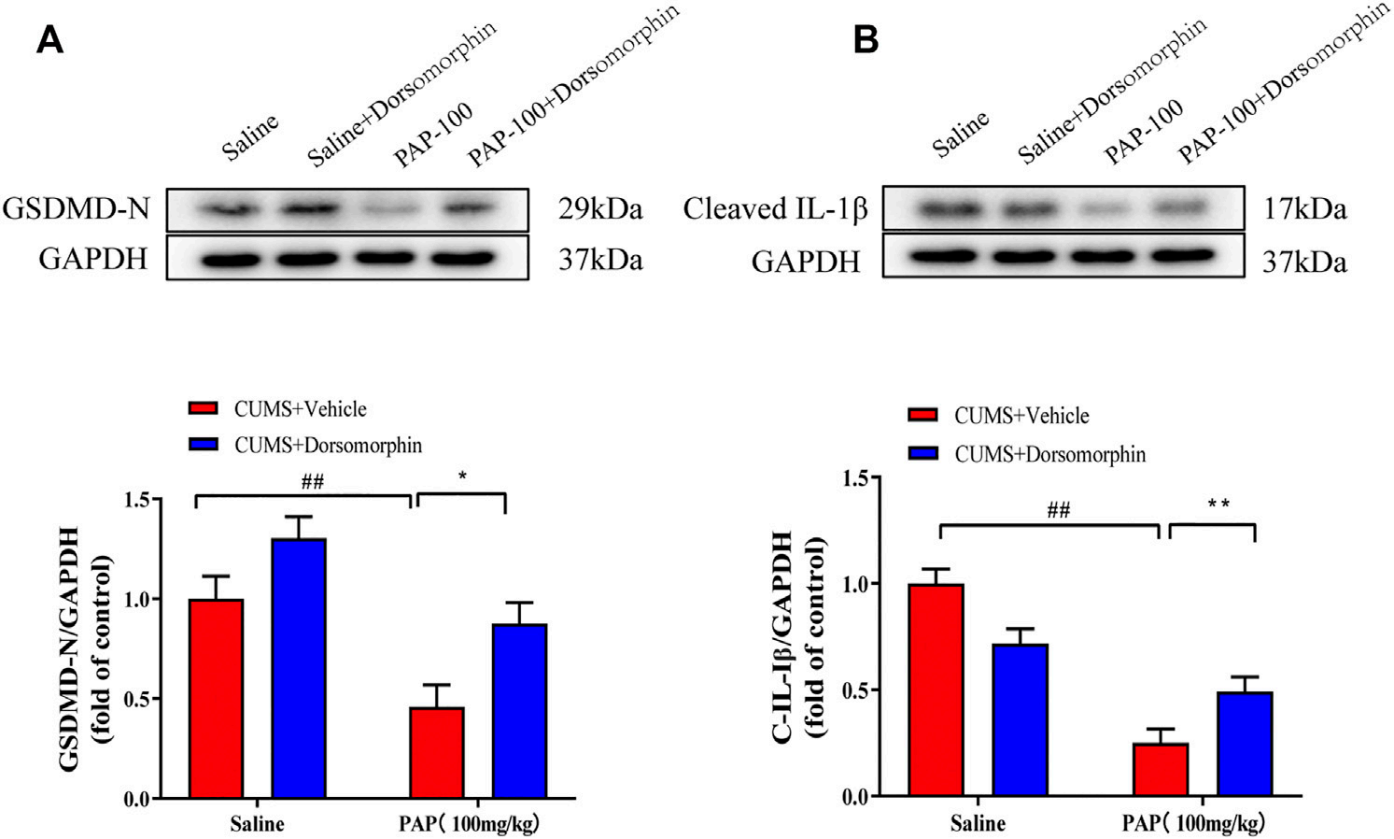
Role of AMPK in the attenuation of PAP on CUMS-induced protein changes**.** The expressions of GSDMD-N **(A)** and Cleaved-IL-1β **(B)** in hippocampal tissues. ##*p* < 0.01 vs. CUMS + Vehicle, *p < 0.05, and **p < 0.01 vs. CUMS + Vehicle + PAP group.

In addition, as shown in Figure 7, the expression levels of GSDMD-N (Figure 7A) and Cleaved-IL-1β (Figure 7B) were remarkably downregulated in the CUMS + PAP (100 mg/kg) group compared with those in the CUMS + Vehicle group (*p* < 0.01). The exposure to CUMS + Dorsomorphin + PAP (100 mg/kg) markedly restored the GSDMD-N expression compared with that in the CUMS + PAP (100 mg/kg) group (*p* < 0.05). These results demonstrated the pivotal role of AMPK in PAP-regulated depression.

## Discussion

In the present study, we used the CUMS-induced mouse model to investigate the effect of PAP on depression-like behaviors and its potential underlying mechanisms. Our data demonstrated that with a 2-week treatment of PAP, the depression-like behaviors of mice were significantly attenuated. Moreover, the neuronal loss was decreased, and the dendritic spine density was restored. Additionally, the protein expression of key regulators in the AMPK/Sirt1/NF-κB/NLRP3 pathway also can be altered with the treatment of PAP. To further explore the mechanism of how PAP exerts antidepressant properties, the Sirt1 inhibitor EX-527 and AMPK inhibitor dorsomorphin were induced in the second and third series of animal experiments. Interestingly, we found that by blocking the expressions of Sirt1 and AMPK, the therapeutic efficacy of PAP was altered, suggesting that AMPK and Sirt1 play critical roles in the PAP-relieved depression.

PAP, purified and isolated from pilose antler, is acknowledged as an anti-inflammatory and anti-oxidative candidate for intervening in various types of diseases (Bai et al., 2017; Chunhui et al., 2017; Ma et al., 2017; Wang et al., 2019). Previous studies demonstrated that PAP not only affects the expression of inflammatory cytokines, e.g., tumor necrosis factor-a (TNF-a), IL-6, and IL-1β, but also manipulates several inflammation-related signaling pathways, e.g., the NF-κB pathway (Liu et al., 2017), TGF-β/SMAD pathway (Xu et al., 2021), or EGF/EGFR signaling pathway (Chunhui et al., 2017). For instance, with the treatment of PAP, the production of inflammatory cytokines was significantly inhibited in osteoblastic cells; meanwhile, the levels of catalase (SOD) and malondialdehyde (MDA) were also decreased. Moreover, nuclear erythroid factor 2-related factor 2 (Nrf2)2/heme oxygenase-1(HO-1) signaling can be stimulated, whereas the NF-κB pathway can be inhibited with PAP treatment. In addition, the knockdown of EGF somehow compromises the PAP-induced cytoprotection (Chunhui et al., 2017). Our previous study also demonstrated that PAP has a similar positive therapeutic effect in LPS-induced nucleus pulposus cells (Dong et al., 2018). However, up to now, the effect of PAP on the depression model remains largely unknown.

The therapeutic effect of PAP on depression-like behaviors and its regulatory role on protein expressions of NF-κB, NLRP3, ASC, Caspase-1, GSDMD-N, Cleaved-IL-1β, and Cleaved-IL-18, etc., paved the way for us to understand PAP and its effect on pyroptosis. Pyroptosis is identified to be a programmed cell death regulated by inflammation (Xue et al., 2019). It is acknowledged that in the progression of pyroptosis, the inflammasome complex is activated and assembled, which can recruit ASC and Caspase-1 (Vande Walle and Lamkanfi, 2016; Sun et al., 2019). The auto-proteolytic cleavage of Caspase-1 triggers the proteolysis of the inflammatory cytokines such as IL-1β and IL-18 to form their bioactive substances (Pandey et al., 2021). The promotion of the NLRP3 inflammasome activates GSDMD-N, consequently leading to pyroptosis-triggered cell death (Guo et al., 2019). Slowik et al. elicited that the suppression of pyroptosis was an efficient therapeutic strategy for preventing depression (Slowik et al., 2018). It has been proposed that the Caspase-1 inhibitor VX765 suppressed the expression of GSDMD and its cleavage form GSDMD-N, which diminished microglia activation and protein cognitive impairment (Xu et al., 2019). Moreover, NLRP3-mediated pyroptosis can be regulated by NF-κB, which participates in numerous inflammatory reactions by the transcription of pro-inflammatory factors (He et al., 2016). The acetylation of NF-κB is the pivotal activator of NLRP3/GSDMD-modulated pyroptosis (Teng et al., 2020). In our study, with the treatment of PAP, the expressions of Ac-NF-κB, NF-κB, NLRP3, ASC, Ac-Caspase-1, Caspase-1, GSDMD-N, Cleaved-IL-1β, and Cleaved-IL-18 in the hippocampus were significantly downregulated compared with those of the CUMS group, indicating that PAP may have an effect on the whole process of depression-induced pyroptosis.

To further validate the role of PAP on the pyroptosis-related pathway, the expressions of Sirt1 and AMPK were also tested in different groups. Sirt1, as a member of the NAD-dependent sirtuin family, activates the deacetylation of acetyl groups on lysine residues of proteins, thus regulating their biological functions (Nogueiras et al., 2012; Chen et al., 2020). The overexpression of Sirt1 blocks the NLRP3 inflammasome activation and pyroptosis in cadmium-induced nephrotoxicity (Chou et al., 2019). As for the relationship between Sirt1 and NF-κB, studies demonstrated that anxiety-like behavior caused by brain hypoxia can be suppressed by Sirt1 *via* the NF-κB pathway (Fan et al., 2018). Moreover, it was displayed that the Sirt1 inhibitor EX527 or Sirt1 knocking down lentivirus (sh-Sirt1) in the hippocampus ameliorated CUMS-induced depression-like behaviors and the cognitive deficiency (Shen et al., 2019). In addition, the NLRP3 inflammasome is closely associated with the upstream elements including AMPK (Youm et al., 2015). It was illustrated that the AMPK signaling pathway played a critical role in the mediation of hyperglycemia-induced cardiomyocyte pyroptosis (Zhang et al., 2020). Furthermore, NLRP3-mediated pyroptosis controlled by the AMPK cascade was highly related to the neuroprotective activity in cerebral ischemia-reperfusion injury (An et al., 2019). All these studies suggested that Sirt1 and AMPK were involved in NF-κB/NLRP3-mediated pyroptosis. Elbaz et al. also proved that the AMPK/Sirt1 pathway was implicated in the suppression of the inflammatory response and attenuation of behavior dysfunction (Elbaz et al., 2018). In our study, results confirmed that PAP treatment effectively restored the expression levels of p-AMPK and Sirt1 and inhibited the expression levels of Ac-NF-κB, NLRP3, Ac-Caspase-1, GSDMD-N, Cleaved-IL-1β, and Cleaved-IL-1β in the hippocampus. With the application of the Sirt1 selective inhibitor EX-527 and AMPK selective inhibitor dorsomorphin, we confirmed that the AMPK/Sirt1 signaling pathway was related to PAP-mediated depression.

In summary, our experiment demonstrated that PAP ameliorated CUMS-induced depression *via* AMPK/Sirt1/NF-κB/NLRP3-mediated pyroptosis. PAP may be a candidate therapeutic drug for depression, but this needs further research before clinical application.

## Conclusion

In summary, our experiment demonstrated that PAP ameliorated CUMS-induced depression *via* AMPK/Sirt1/NF-κB/NLRP3-mediated pyroptosis. PAP may be a candidate therapeutic drug for depression, but this needs further research before clinical application.

## Data Availability

The raw data supporting the conclusions of this article will be made available by the authors, without undue reservation.
