# Supplementary material for: The Protective Effect of Pilose Antler Peptide on CUMS-Induced Depression Through AMPK/Sirt1/NF-κB/NLRP3-Mediated Pyroptosis
Source: Front Pharmacol. 2022 Mar 23;13:815413. doi: 10.3389/fphar.2022.815413 (PMC8984150; doi:10.3389/fphar.2022.815413)

**Series 1**

**p-AMPK**


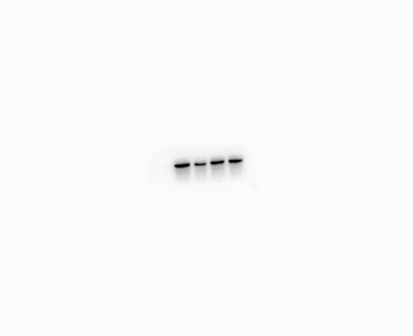


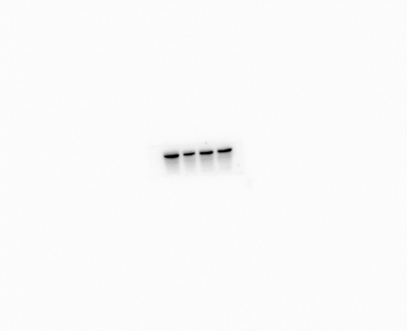


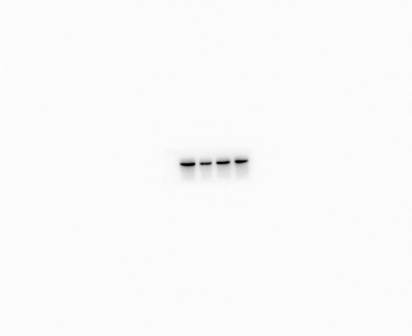


**GAPDH**


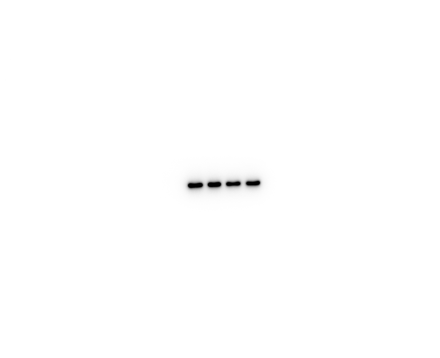


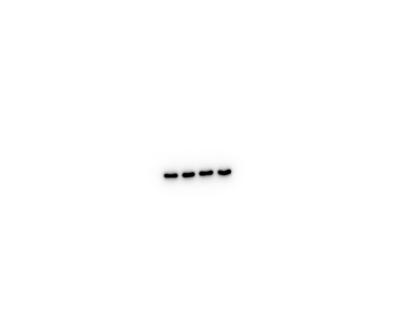


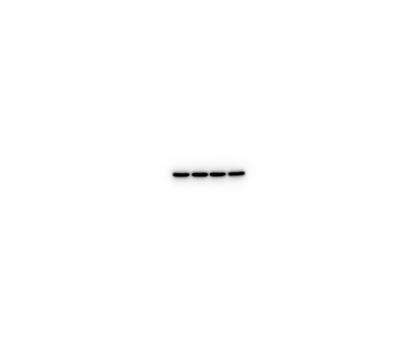


**Sirt1**


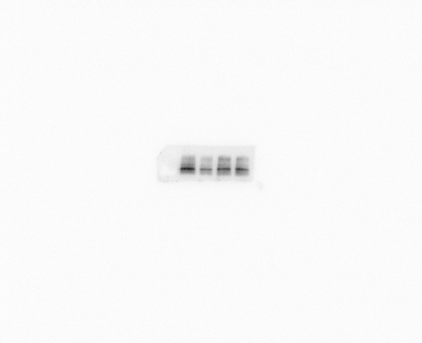


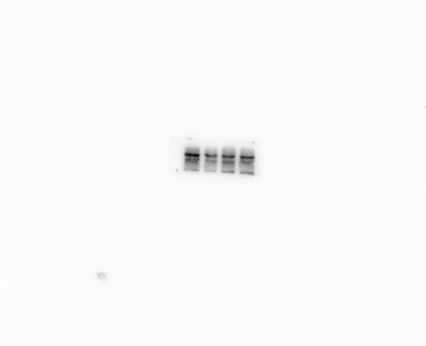


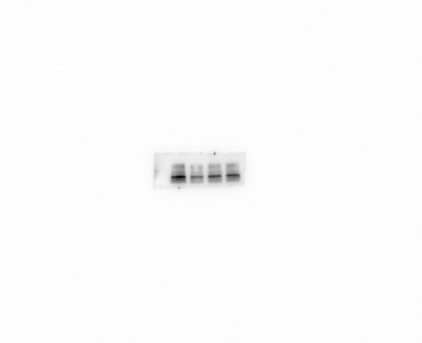


**GAPDH**


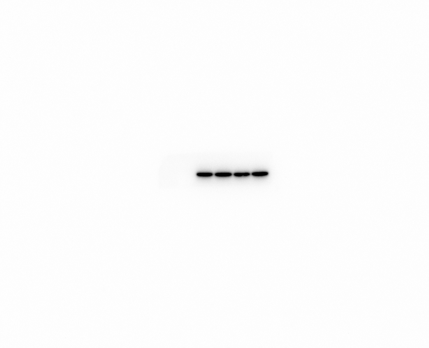


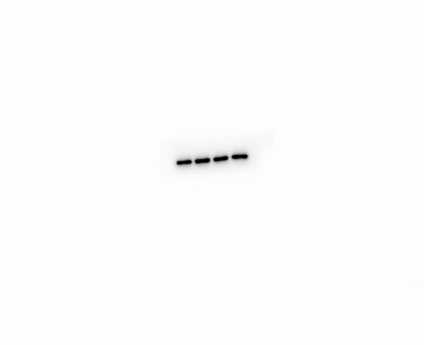


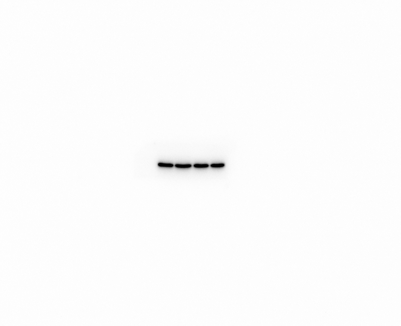


**Ac-NF-κB**


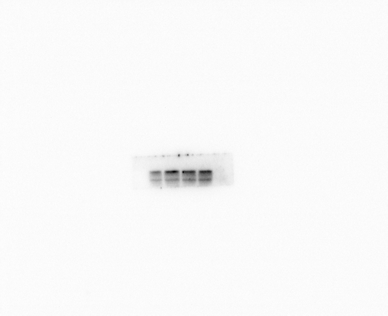


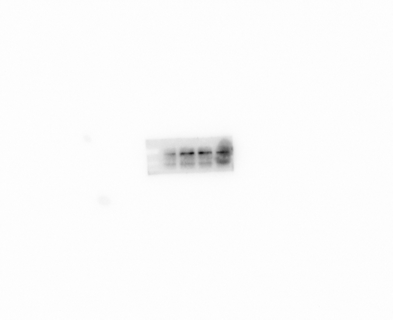


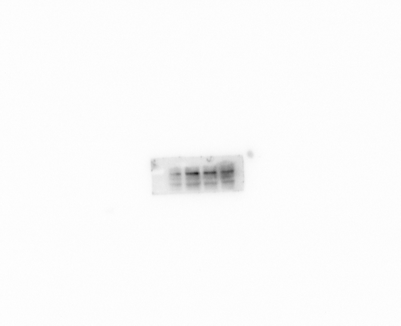


**NF-κB**


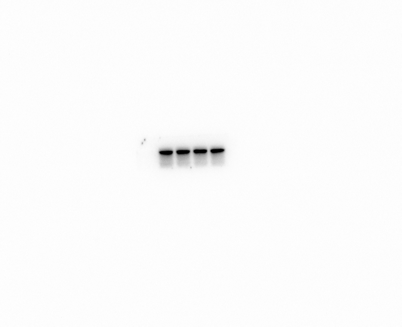


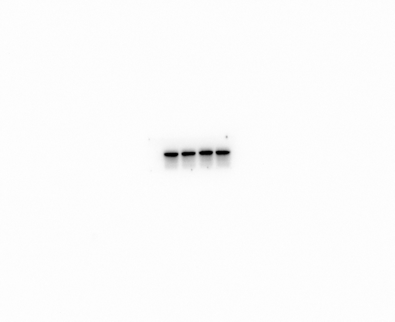


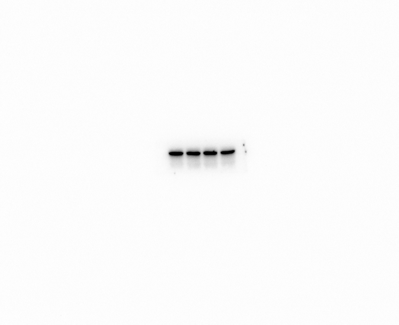


**NLRP3**


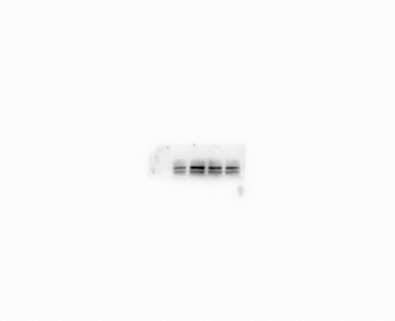


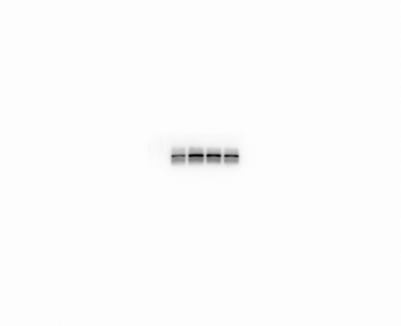


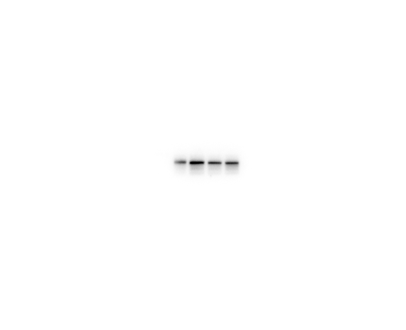


**GAPDH**


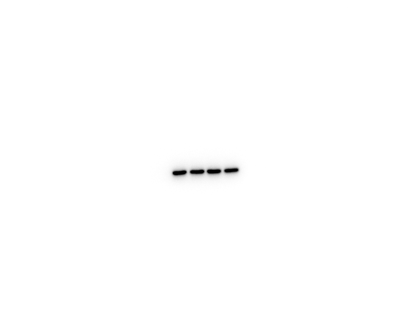


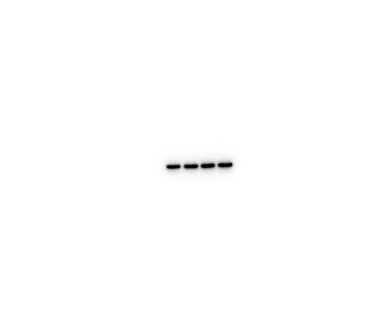


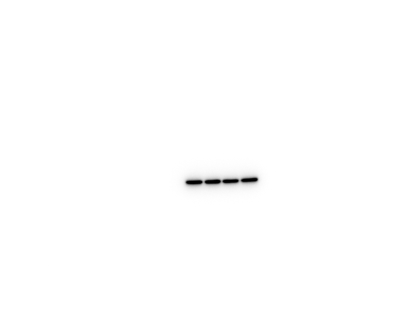


**ASC**


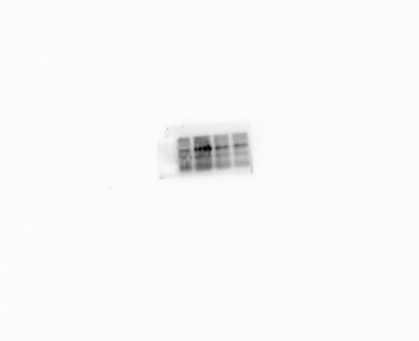


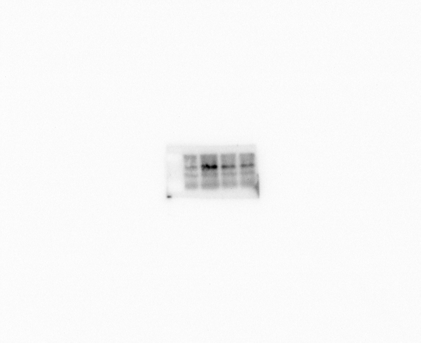


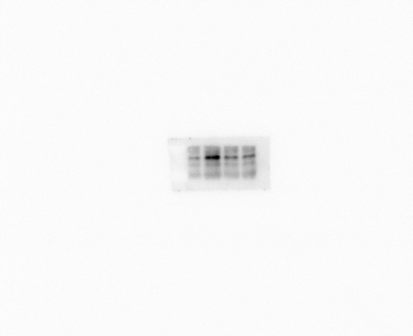


**Ac-Caspase-1**


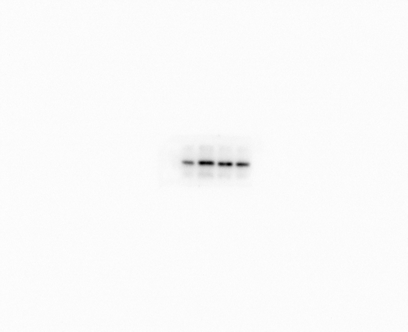


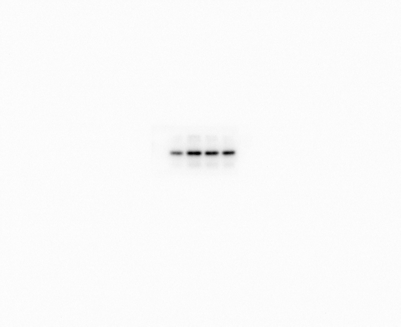


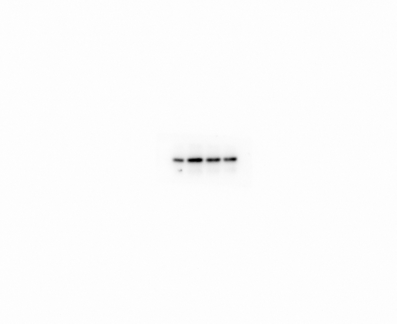


**Caspase-1**


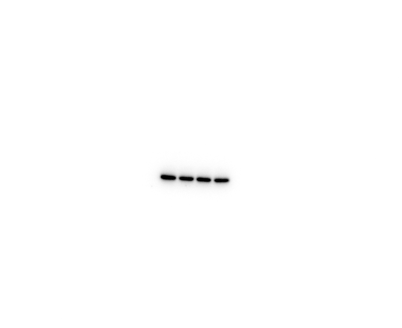


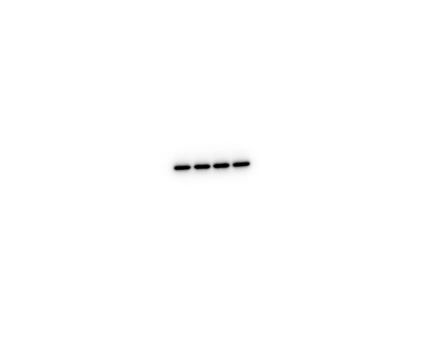


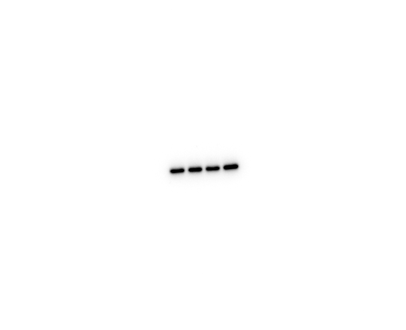


**GSDMD-N**


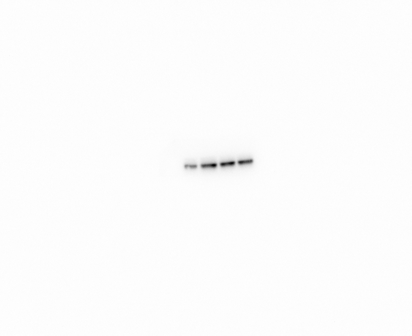


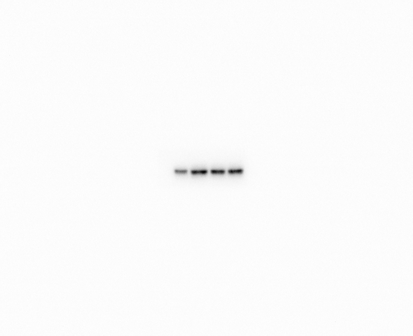


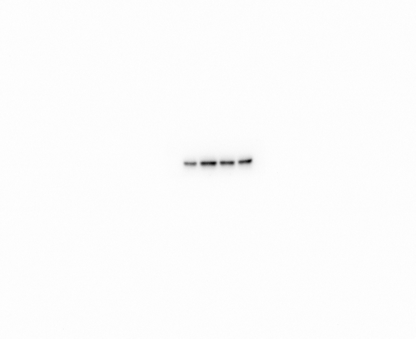


**GAPDH**


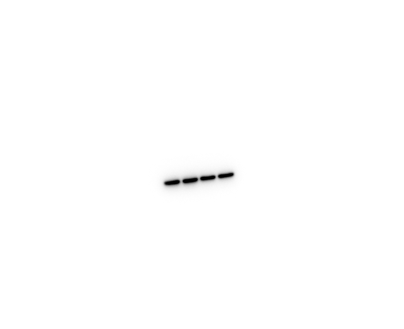


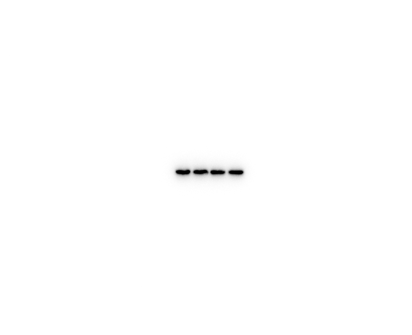


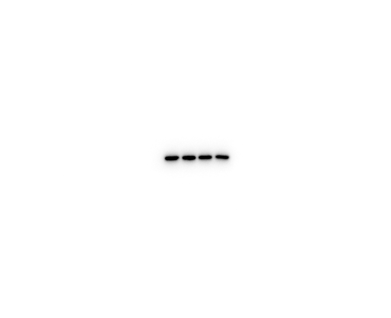


**Cleaved-IL-18**


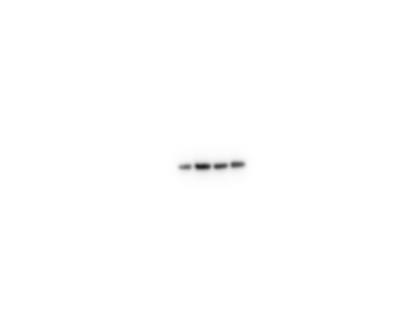


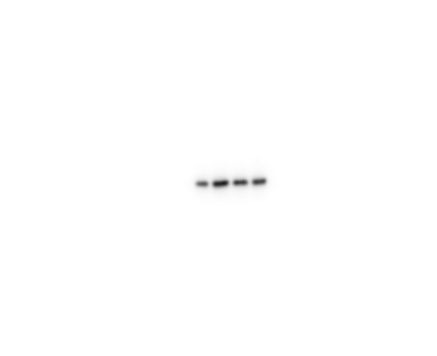


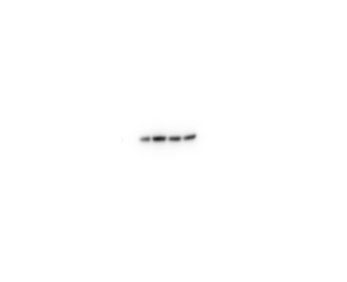


**GAPDH**


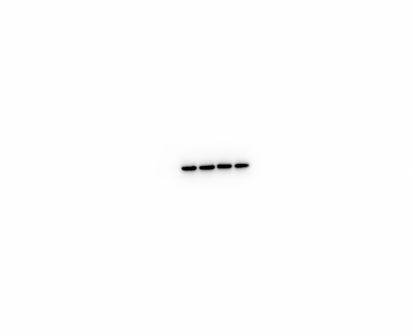


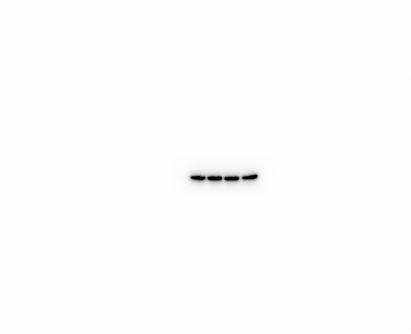


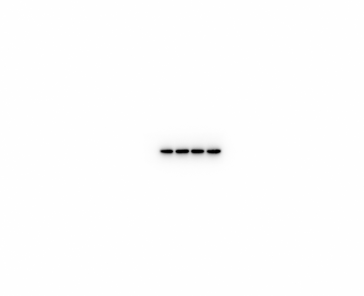


**Cleaved-IL-1β**


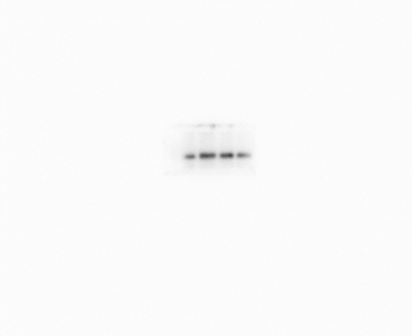


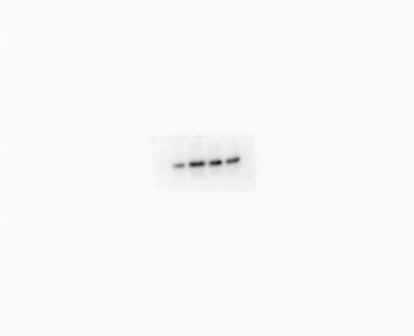


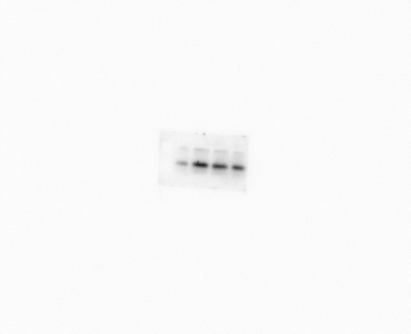


**GAPDH**


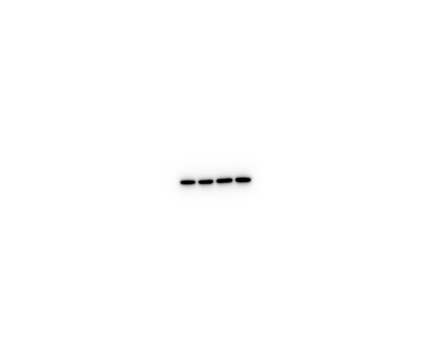


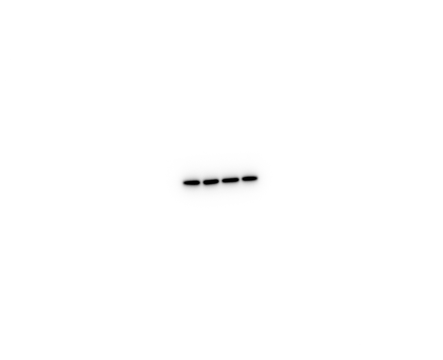


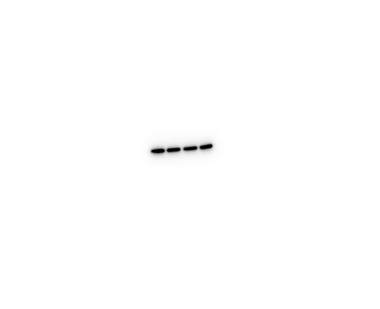

Supplement: Supplementary file 1 [file DataSheet4.docx]
