# Supplementary figures and images for: The Protective Effect of Pilose Antler Peptide on CUMS-Induced Depression Through AMPK/Sirt1/NF-κB/NLRP3-Mediated Pyroptosis
Source: Front Pharmacol. 2022 Mar 23;13:815413. doi: 10.3389/fphar.2022.815413 (PMC8984150; doi:10.3389/fphar.2022.815413)

**Series 3**

**Cleaved-IL-1β**


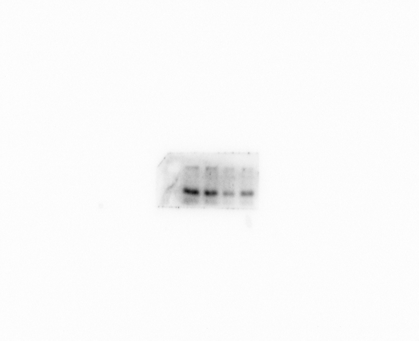


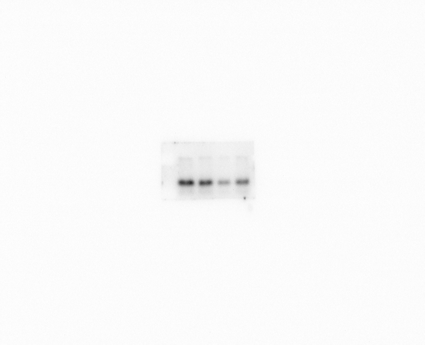


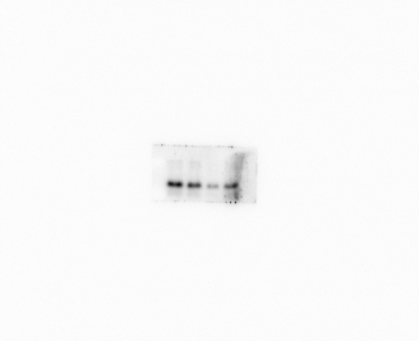


**GAPDH**


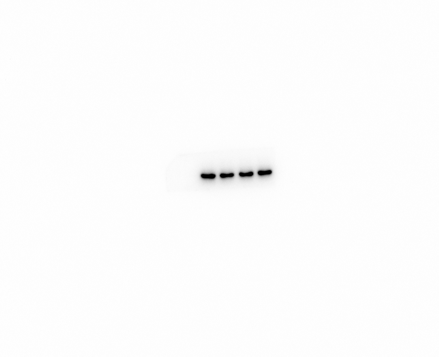


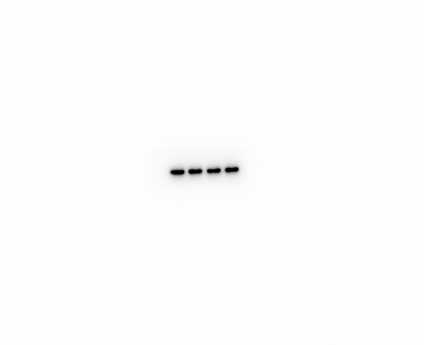


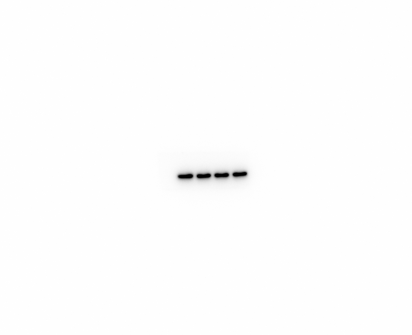


**GSDMD-N**


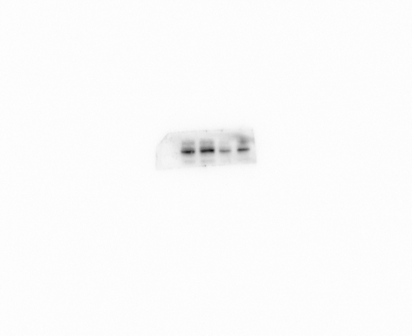


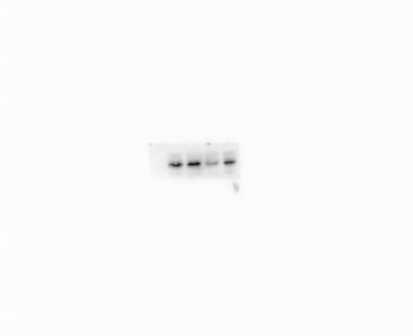


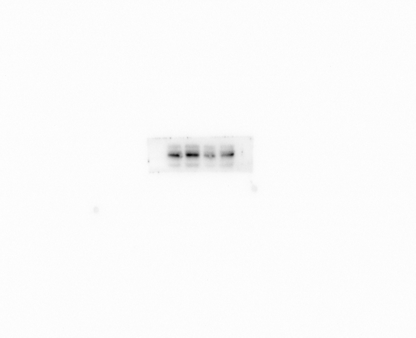


**GAPDH**


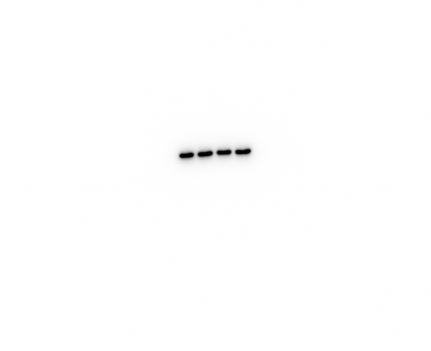


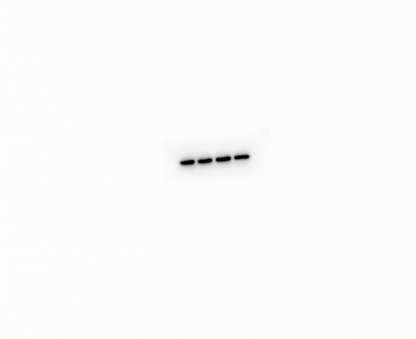


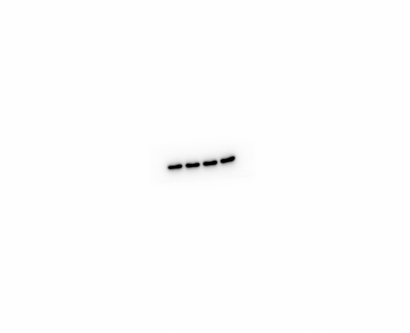

Supplement: Supplementary file 2 [file DataSheet6.docx]

**Series 2**

**Ac-Caspase-1**


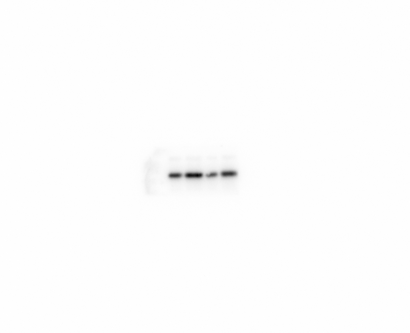


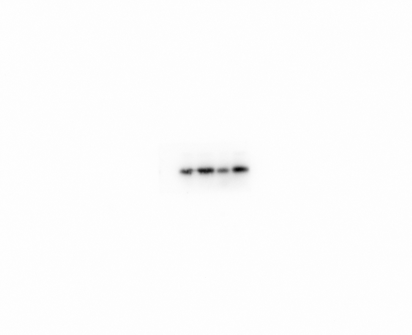


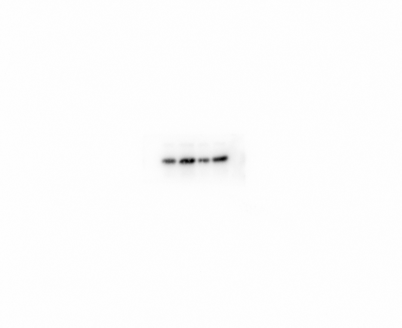


**Caspase-1**


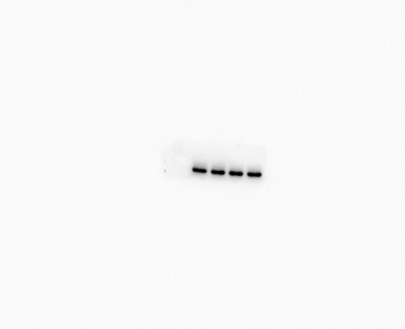


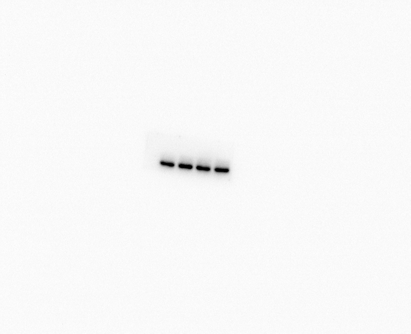


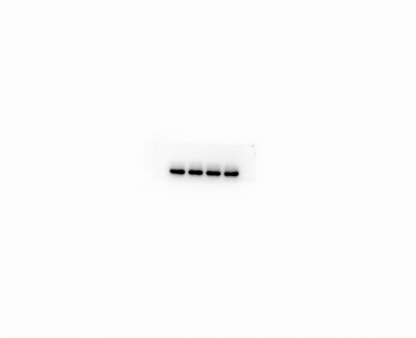


**Cleaved-IL-1β**


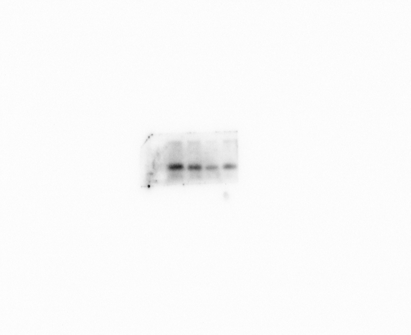


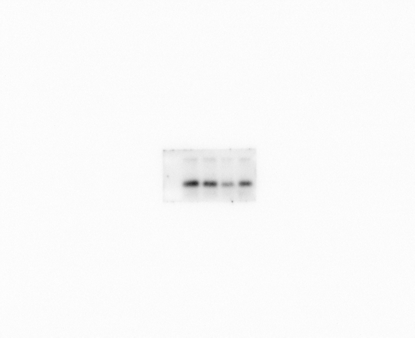


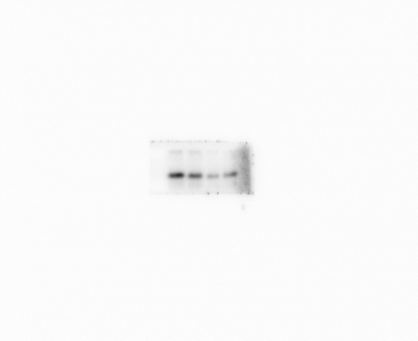


**GSDMD-N**


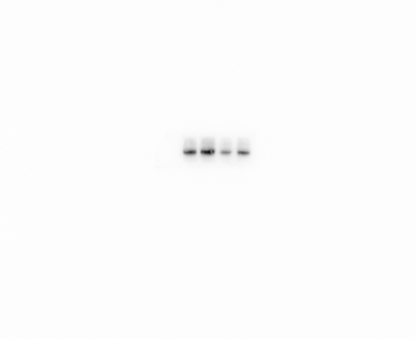


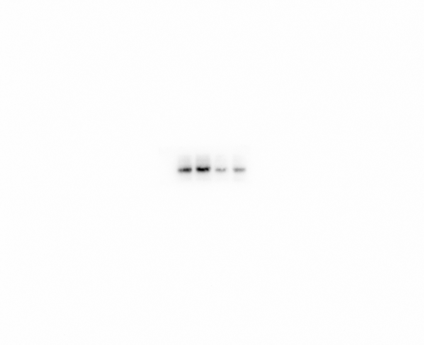


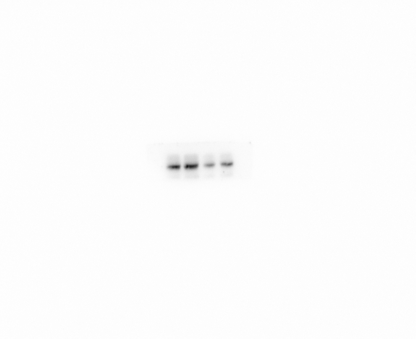


**GAPDH**


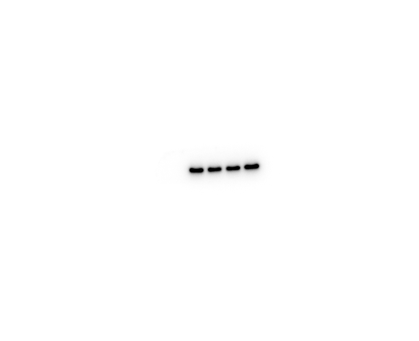


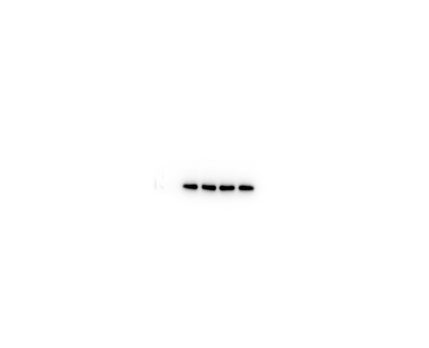


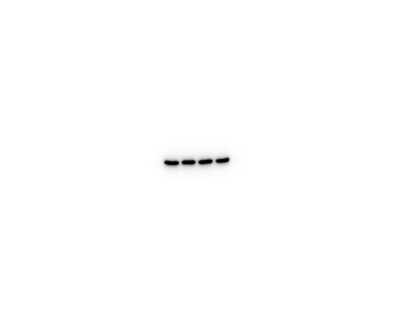

Supplement: Supplementary file 4 [file DataSheet5.docx]
