# Supplementary material for: The Protective Effect of Pilose Antler Peptide on CUMS-Induced Depression Through AMPK/Sirt1/NF-κB/NLRP3-Mediated Pyroptosis
Source: Front Pharmacol. 2022 Mar 23;13:815413. doi: 10.3389/fphar.2022.815413 (PMC8984150; doi:10.3389/fphar.2022.815413)
Supplement: Supplementary file 3 [file Presentation1.pdf]

Supplementary Material

1 Supplementary Figures

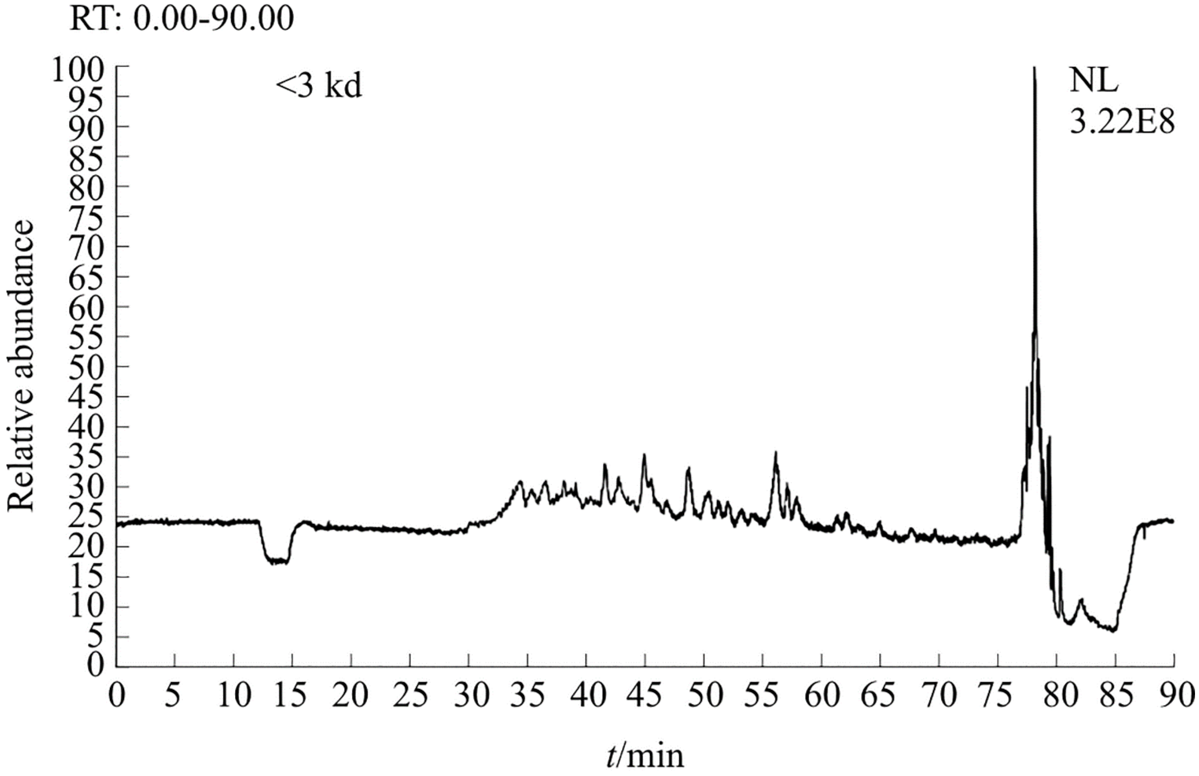

Fig. S1. TIC chromatograms of peptides in Pilose Antler by LC-LTQ/Orbitrap MS.

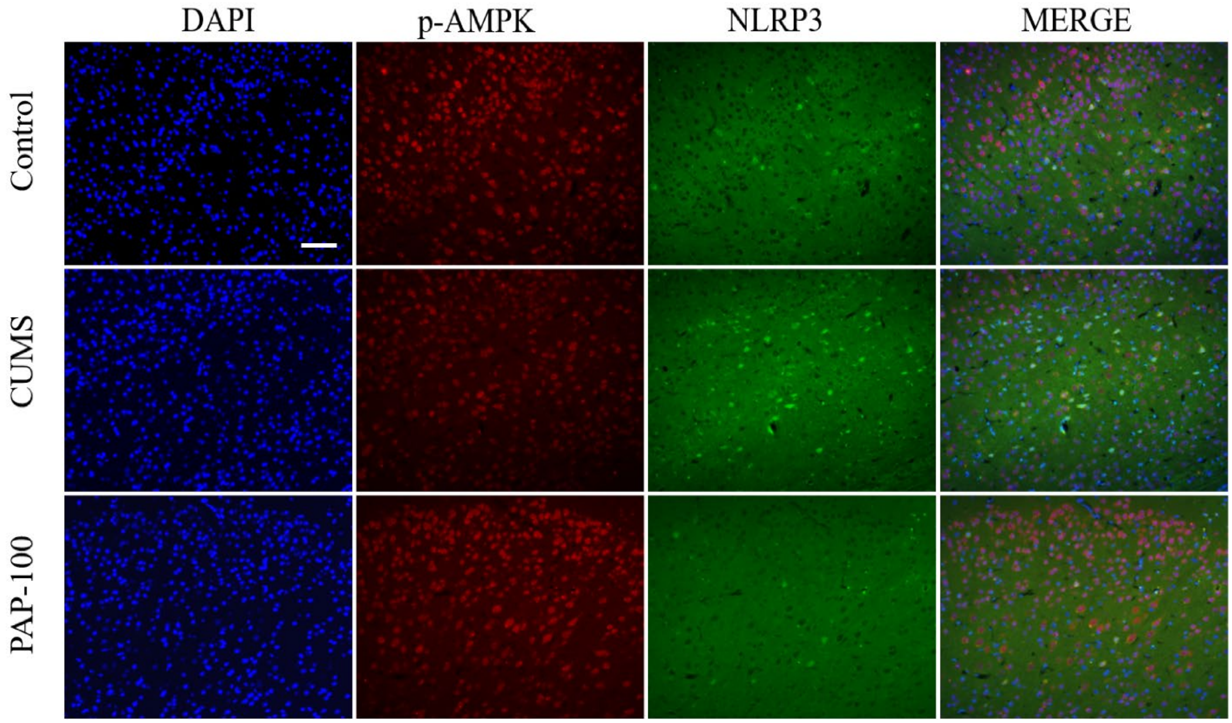

Fig. S2. Immunofluorescence staining of p-AMPK and NLRP3 in the hippocampus. Co-localization of p-AMPK and NLRP3 in the hippocampus of control, CUMS, and CUMS treated with 100 mg/kg PAP. Scale bar: 50μm.

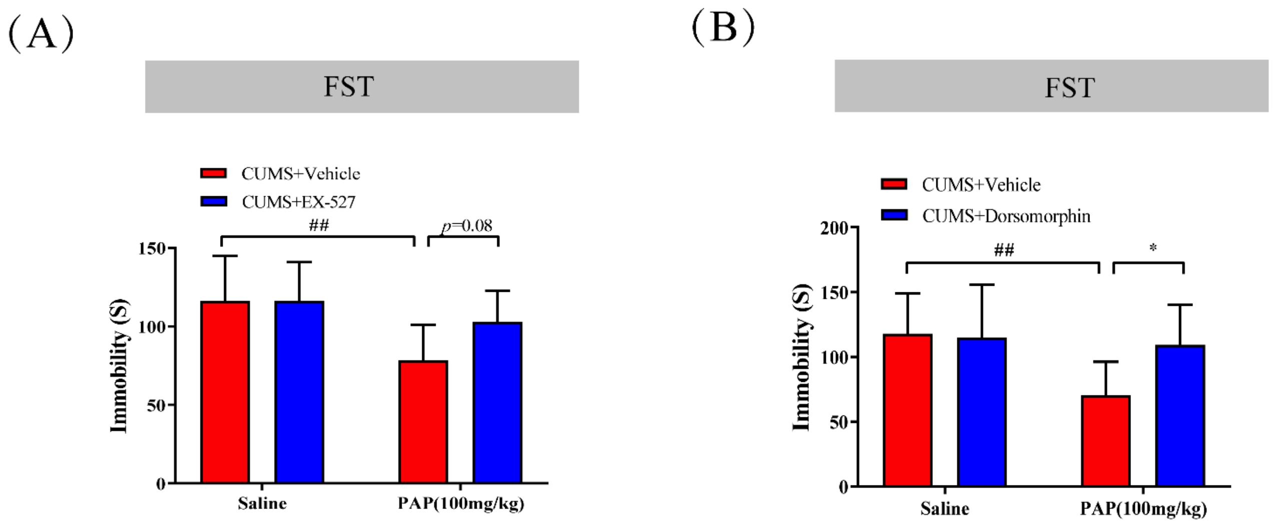

Fig. S3. The effect of PAP in FST. (A) FST was measured in four groups: CUMS + Vehicle, CUMS + EX-527, CUMS + Vehicle + PAP (100mg/kg) and CUMS + EX-527 + PAP (100mg/kg). (B) FST was measured in four groups: CUMS + Vehicle, CUMS + Dorsomorphin, CUMS + Vehicle + PAP (100mg/kg) and CUMS + Dorsomorphin + PAP (100mg/kg).
